# Nerve growth factor receptor (Ngfr) induces neurogenic plasticity by suppressing reactive astroglial Lcn2/Slc22a17 signaling in Alzheimer’s disease

**DOI:** 10.1038/s41536-023-00311-5

**Published:** 2023-07-10

**Authors:** Tohid Siddiqui, Mehmet Ilyas Cosacak, Stanislava Popova, Prabesh Bhattarai, Elanur Yilmaz, Annie J. Lee, Yuhao Min, Xue Wang, Mariet Allen, Özkan İş, Zeynep Tansu Atasavum, Natalia Rodriguez-Muela, Badri N. Vardarajan, Delaney Flaherty, Andrew F. Teich, Ismael Santa-Maria, Uwe Freudenberg, Carsten Werner, Giuseppe Tosto, Richard Mayeux, Nilüfer Ertekin-Taner, Caghan Kizil

**Affiliations:** 1 German Center for Neurodegenerative Diseases (DZNE) within Helmholtz Association, 01307 Dresden, Germany; 2 Department of Neurology, Columbia University Irving Medical Center, New York, NY 10032 USA; 3 Taub Institute for Research on Alzheimer’s Disease and the Aging Brain, Columbia University Irving Medical Center, New York, NY 10032 USA; 4 The Gertrude H. Sergievsky Center, College of Physicians and Surgeons, Columbia University, 630 West 168th Street, New York, NY 10032 USA; 5 Department of Neuroscience, Mayo Clinic Florida, Jacksonville, FL 32224 USA; 6 Department of Quantitative Health Sciences, Mayo Clinic Florida, Jacksonville, FL 32224 USA; 7 Department of Pathology and Cell Biology, Columbia University Irving Medical Center, New York, NY 10032 USA; 8 Facultad de Ciencias Experimentales, Universidad Francisco de Vitoria, Edificio E, 28223, Pozuelo de Alarcon, Madrid, Spain; 9 Leibniz-Institut für Polymerforschung Dresden e.V., Hohe Str. 6, D-01069 Dresden, Germany; 10 Cluster of Excellence Physics of Life, TU Dresden, D-01307 Dresden, Germany; 11 Department of Psychiatry, College of Physicians and Surgeons, Columbia University, 1051 Riverside Drive, New York, NY 10032 USA; 12 Department of Neurology, Mayo Clinic Florida, Jacksonville, FL 32224 USA; 13 Present Address: Neuron D GmbH, Tatzberg 47, 01307 Dresden, Germany

**Keywords:** Adult neurogenesis, Neural stem cells, Regeneration and repair in the nervous system

## Abstract

Neurogenesis, crucial for brain resilience, is reduced in Alzheimer’s disease (AD) that induces astroglial reactivity at the expense of the pro-neurogenic potential, and restoring neurogenesis could counteract neurodegenerative pathology. However, the molecular mechanisms promoting pro-neurogenic astroglial fate despite AD pathology are unknown. In this study, we used APP/PS1dE9 mouse model and induced Nerve growth factor receptor (*Ngfr*) expression in the hippocampus. Ngfr, which promotes neurogenic fate of astroglia during the amyloid pathology-induced neuroregeneration in zebrafish brain, stimulated proliferative and neurogenic outcomes. Histological analyses of the changes in proliferation and neurogenesis, single-cell transcriptomics, spatial proteomics, and functional knockdown studies showed that the induced expression of *Ngfr* reduced the reactive astrocyte marker Lipocalin-2 (Lcn2), which we found was sufficient to reduce neurogenesis in astroglia. Anti-neurogenic effects of Lcn2 was mediated by Slc22a17, blockage of which recapitulated the pro-neurogenicity by Ngfr. Long-term Ngfr expression reduced amyloid plaques and Tau phosphorylation. Postmortem human AD hippocampi and 3D human astroglial cultures showed elevated LCN2 levels correlate with reactive gliosis and reduced neurogenesis. Comparing transcriptional changes in mouse, zebrafish, and human AD brains for cell intrinsic differential gene expression and weighted gene co-expression networks revealed common altered downstream effectors of NGFR signaling, such as *PFKP*, which can enhance proliferation and neurogenesis in vitro when blocked. Our study suggests that the reactive non-neurogenic astroglia in AD can be coaxed to a pro-neurogenic fate and AD pathology can be alleviated with Ngfr. We suggest that enhancing pro-neurogenic astroglial fate may have therapeutic ramifications in AD.

## Introduction

The generation of new neurons in adulthood reduces with vertebrate phylogeny^1–4^. Neurogenic regions are spatially restricted in mammals, which limits the addition of new neurons to the existing circuitry^5^. Generation and circuit integration of new neurons contribute to the resilience and cognitive abilities of the brain^6–9^, which could offset late-onset neurodegeneration^10–13^. Neurogenesis reduces in Alzheimer’s disease (AD) patients^14,15^, suggesting an intriguing possibility that enhancing the neurogenic plasticity of the brain could improve the age-related neurodegenerative outcomes. However, the molecular mechanisms of how neural progenitor cells with astroglial identity could be tweaked to become pro-neurogenic despite the prevalent disease pathology that imposes a reactive astroglial state are unknown.

The heterogeneity of the neurogenic ability across vertebrates is vast. Adult teleost fish such as zebrafish has a widespread regenerative ability, which is unparalleled in the vertebrate clades^16,17^. Astroglia acting as neural stem/progenitor cells are one of the key cell types for neurogenic output^18^. We previously generated an adult AD zebrafish model and found that a complex set of cellular crosstalk between astroglia and other cell types that leads to enhanced neurogenesis despite the prevalent disease pathology^19–22^. This model has strong parallelism to human AD brains in terms of molecular programs affected by AD pathology^23^, served as a pre-clinical tool for drug screening^24–26^ and helped identify the cellular functions of genes associated with AD in humans^27,28^. The pathology-induced pro-neurogenic and neuro-regenerative ability of astroglia in zebrafish in AD-like scenarios rely on set of molecular mechanisms^29,30^, one of which is the pro-neurogenic activity through the expression of nerve growth factor receptor (*ngfra*)^19,31^. *Ngfr* is not detected in mouse hippocampal astroglia^32^, and we hypothesized that induced expression of *Ngfr* could alter the neurogenic properties of astroglia under the prevalent AD pathology in mouse brains. In this study, we show that Ngfr signaling, when activated in the hippocampus of an AD mouse model, reduces reactive gliotic state through suppressing Lcn2/Slc22a17 signaling and enhances pro-neurogenic fate of astroglia. We provide a mechanistic and epistatic link between Ngfr activity and downstream transcriptional regulation and phenotypic outcomes including AD pathological hallmarks by using in vitro 3D human neurogenesis assay, large human AD cohorts, and cell intrinsic differential gene expression analyses. Our results identified a mechanism by which astroglia could be coaxed to pro-neurogenic fate that modulates the pathogenesis of Alzheimer’s disease.

## Results

### Viral transduction and targeting of astroglia in the mouse dentate gyrus

**Fig. 1:**
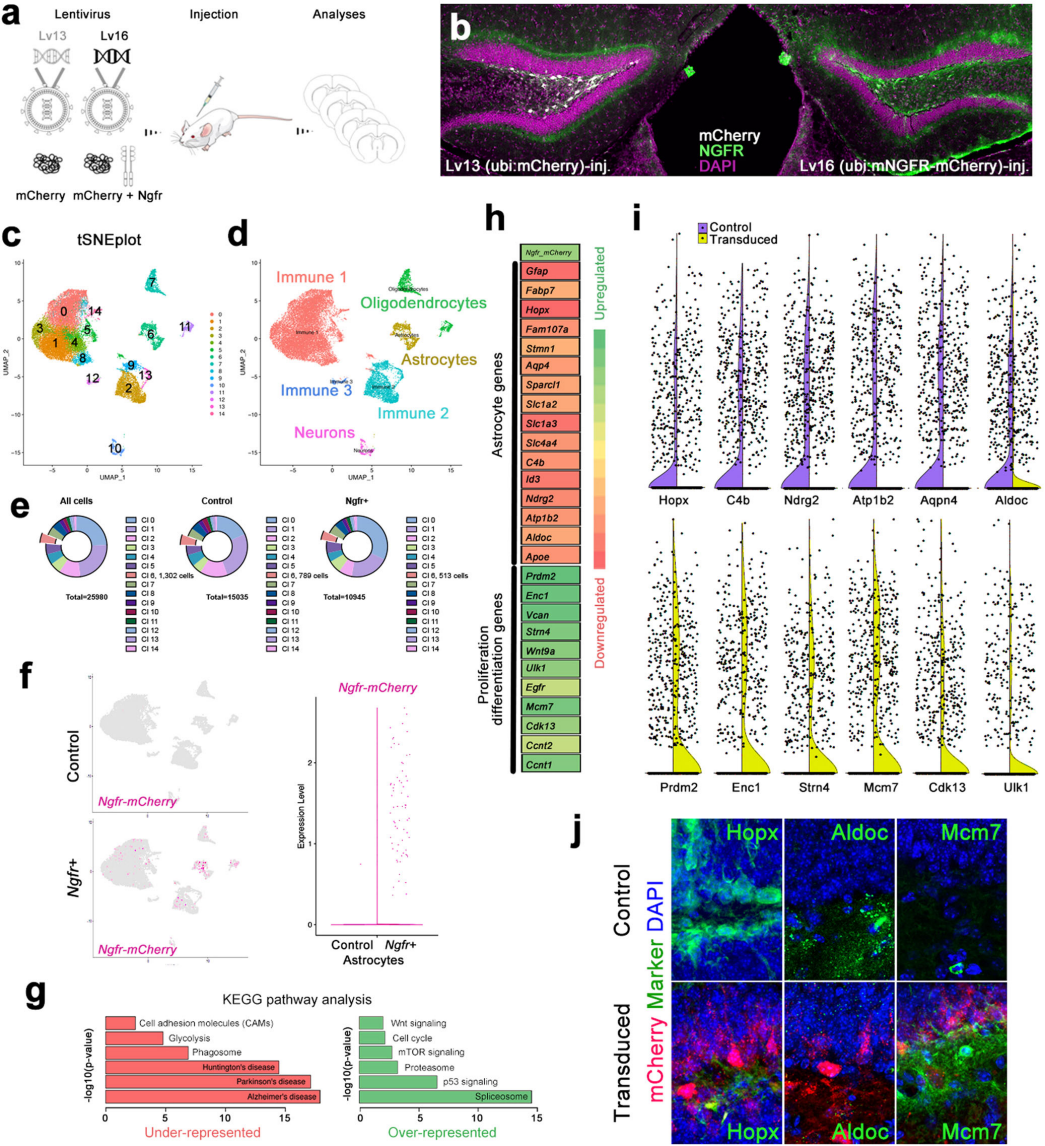
NGFR enhances proliferative and neurogenic markers in dentate gyrus (DG) astrocytes. **a** Schematic strategy for expression lentiviruses: Lv13: mCherry control; Lv16: Ngfr-mCherry. **b** Cross section images for DGs transduced with Lv13 and Lv16 and immunostained for mCherry, NGFR with DAPI counterstain. Note NGFR expression in the subgranular zone (SGZ) after Lv16 transduction. **c** Single cell transcriptomics tSNE plot from dissected DGs after Lv13 and Lv16 transduction. **d** tSNE plots indicating cell types. **e** Number of cells sequenced and their distribution to individual cell types. **f** tSNE plots from Lv13 and Lv16 transduction showing the expression of Ngfr-mCherry, which is detectable only after Lv16. **g** KEGG pathway analyses on astrocytic population, showing downregulated and upregulated pathways. **h** A heat map for selected differentially expressed genes (DEGs) after Lv16. Astrocyte markers are downregulated, and proliferation/neurogenesis markers are upregulated**. i** Violin plots for selected genes: purple: control (Lv13); yellow (Lv16). **j** Immunostaining for validating the DEGs in control and Lv16-transduced DG. Scale bars equal 50 μm.

We generated lentiviral constructs that code for mCherry (control, Lv13) and mCherry with mouse *Ngfr* (Lv16) to express *Ngfr* in the astroglia of the subgranular zone (SGZ) of mouse dentate gyrus (DG) (Supplementary Fig. 1a, b). The injection paradigm combined with the enhanced efficiency of the viral particles for targeting astroglia as documented before^33^, our transduction largely targets Gfap+ astroglia, yet other cell types including oligodendrocytes and microglia are also transduced (Supplementary Fig. 1c, d). Microinjection into mouse SGZ (Fig. 1a) results in targeting and transduction of DG (Fig. 1b and Supplementary Fig. 2). A portion of S100β-positive astrocytes were also transduced (Supplementary Fig. 3). These cells were Gfap+, and we did not detect S100β + /Gfap- astroglia transduced (Supplementary Fig. 3). In control DG, Ngfr is expressed at the periphery of the DG but not in SGZ, while Lv16 transduction leads to ectopic expression (Fig. 1b and Supplementary Fig. 2). The expressed receptor is functional as one of the Ngfr ligands - BDNF - increases the proliferation of mouse DG astrocytes only when Ngfr is expressed (Supplementary Fig. 1e (2D cultures), f (3D cultures)).

### Single cell transcriptomics reveals altered molecular programs in astroglia after Ngfr signaling

To determine the altered molecular programs after *Ngfr* expression in astroglia, we performed single-cell transcriptomics by dissecting the DG from Lv13 and Lv16-transduced wild-type mouse brains (Fig. 1c–f and Supplementary Fig. 4). In total, 25,980 cells were sequenced with total 283.8 million reads, and control (Lv13) and Ngfr+ (Lv16) groups had similar distribution of nFeature (genes), nCount (reads per cell), %mito (percentage of reads belonging to mitochondrial genes), and %ribo (percentage of reads belonging to ribosomal genes) (Supplementary Fig. 4a, b). Average number of reads per cell was 10,923 (75-percentile: 13,578) and average number of genes per cell were 2791 (75-percentile: 3294) (Supplementary Fig. 4b, c). Cell spread on UMAP plots showed overlap between Lv13 and Lv16 group, indicating that all cell types can be identified in both groups comparably (Supplementary Fig. 4d). We identified 15 cell clusters representing the neuronal, immune, astroglial and oligodendroglial cells with their canonical markers determined by violin plots and heat maps (Fig. 1c, d and Supplementary Fig. 4e–g). In total, we sequenced 789 astroglia in control and 513 astroglia in Ngfr+ samples (Fig. 1e). To determine if the power of astroglial cluster was sufficient to perform the subsequent analyses, we used SCOPIT power calculation tool^34^ with the following parameters: minimum 10 cells per cluster must be sequenced, required probability of success 0.99, frequency of the rarest cell population 6%, at least 2 subpopulations of cells can be identified. We found that 328 cells must exist in a cluster for reliable statistical analyses, which we obtained in both samples. Clustering the cell types and plotting Lv16-dependent expression of Ngfr-mCherry (adding the fusion transcript as a synthetic read template that determined the expression of the entire transgene, Fig. 1f and Supplementary Fig. 4h) showed that Lv16 transduction can target astroglia (34.7% of this cell population) as well as microglia (1.3% of this cell population), neurons (2.9% of this cell population) oligodendrocytes (6.33% of this cell population) (Fig. 1f and Supplementary Fig. 3h), noting that some neurons and oligodendrocytes normally express *Ngfr* in control brains (Supplementary Fig. 4h). Control transductions with a control virus (Lv13) expressing only *mCherry* did not show any *Ngfr*-mCherry fusion construct expression (Fig. 1 and Supplementary Fig. 4h).

### Molecular pathways related to proliferation and neurogenesis altered upon Ngfr

To determine the effects of *Ngfr* transduction on astroglia, we performed differentially expressed gene (DEG) (Supplementary Data 1) and pathway analyses (Supplementary Data 2 and 3) within the astroglial cluster (Cluster 6). We found that *Ngfr* expression reduced pathways related to neurodegenerative diseases, including AD, while induced pathways related to sustained homeostasis and growth, such as proteasome, mTOR signaling and cell cycle (Fig. 1g). Analyses of DEGs in astroglia showed a general reduction in astrocytic markers that are associated with reactive or non-neurogenic states (e.g.,: *Gfap, Apoe, Hopx, Ndrg2, Aldoc, Id3*) and an overexpression of genes associated with proliferation and neurogenic differentiation (e.g.,: *Prdm2, Enc1*, *Egfr, Mcm7, Cdk13*) (Fig. 1h–j). This suggested that Ngfr signaling could promote proliferative and pro-neurogenic route in mouse DG astroglia.

### Ngfr increases astroglial proliferation and neurogenesis

**Fig. 2:**
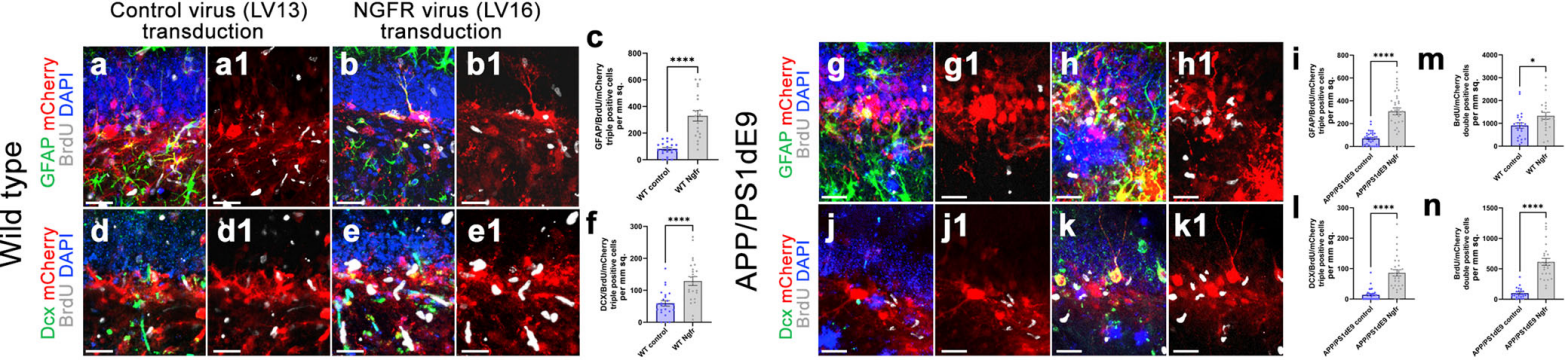
NGFR promotes proliferation of DG astrocytes and neurogenesis in wild type and APP/PS1dE9 model of AD. Immunostaining for GFAP, BrdU and mCherry with DAPI counterstain in Lv13- (**a**) and Lv16- (**b**) SGZ of wild type mice. **a1**, **b1**. BrdU/mCherry channels of a and b. **c** Quantification graph for mCherry-BrdU-GFAP triple positive cells. **d**, **e** Immunostaining for Dcx, BrdU and mCherry with DAPI counterstain in Lv13- (**a**) and Lv16-transduced (**b**) SGZ. **d1**, **e1**. BrdU/mCherry channels of d and e. **f** Quantification graph for mCherry-BrdU-Dcx triple positive cells of wild type mice. Immunostaining for GFAP, BrdU and mCherry with DAPI counterstain in Lv13- (**g**) and Lv16-transduced (**h**) SGZ of APP/PS1dE9 mice. **g1**, **h1**. BrdU/mCherry channels of **g** and **h**. **i** Quantification graph for mCherry-BrdU-GFAP triple positive cells. Immunostaining for Dcx, BrdU and mCherry with DAPI counterstain in Lv13- (**j**) and Lv16-transduced (**k**) SGZ of APP/PS1dE9 mice. **j1**, **k1**. BrdU/mCherry channels of **j** and **k**. **l** Quantification graph for mCherry-BrdU-Dcx triple positive cells. **m** Quantification of BrdU/mCherry double positive cells in wild type mice (control and Ngfr+). **n** Quantification of BrdU/mCherry double positive cells in APP/PS1dE9 mice (control and Ngfr+). Scale bars equal 50 µm. Error bars represent the standard error of the means, with each point representing one section. 3 mice were used.

**Fig. 3:**
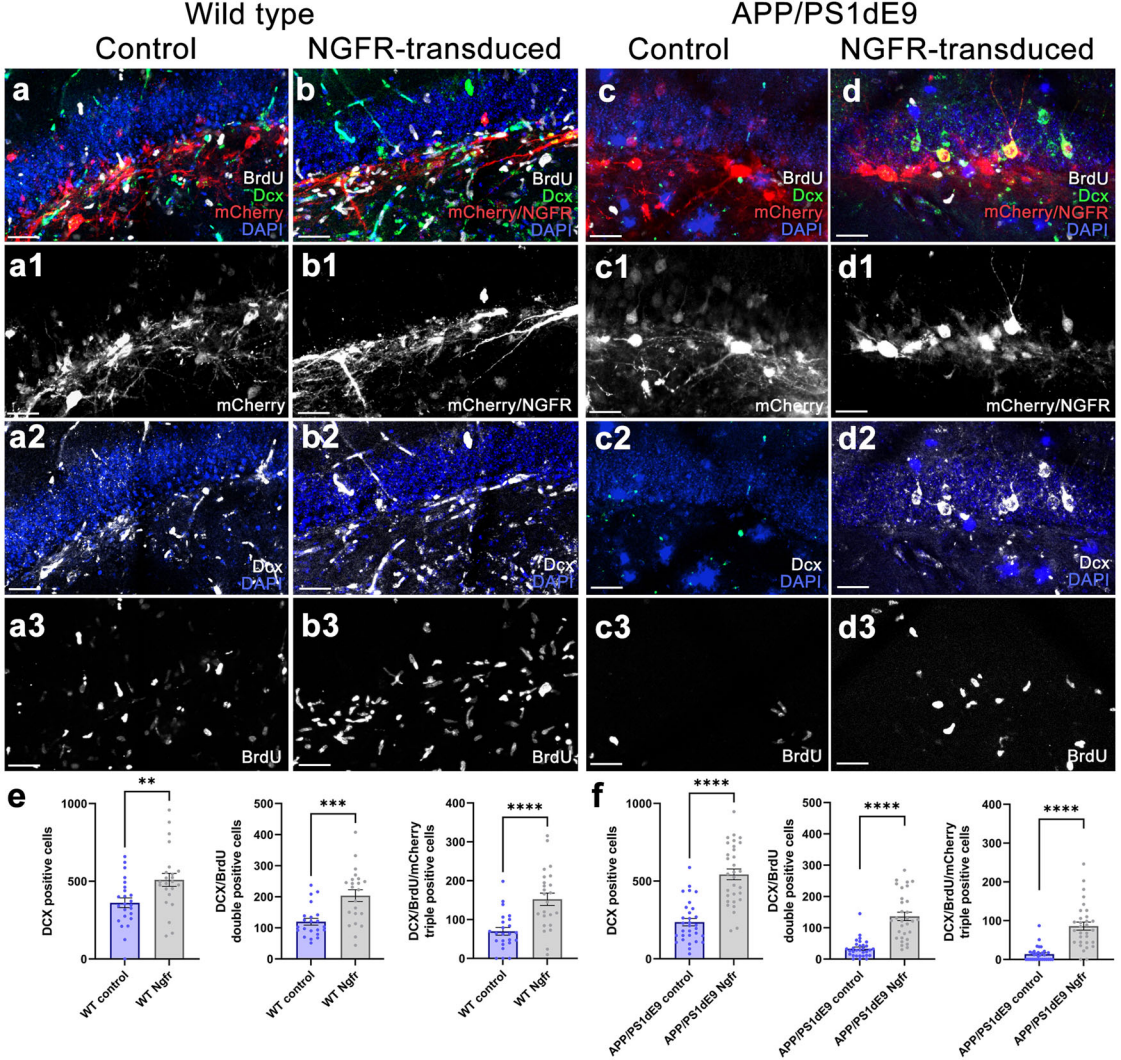
Lv16 transduction enhances neurogenesis in wild type and APP/PS1dE9 mouse model of Alzheimer’s disease. **a** Dcx, mCherry, and BrdU triple immunostaining with DAPI counterstain in Lv13-transduced wild type mouse dentate gyrus. **a1**. mCherry channel of a. **a2**. Dcx and DAPI channel of a. **a3**. BrdU channel of a. **b** Dcx, mCherry, and BrdU triple immunostaining with DAPI counterstain in Lv16-transduced wild type mouse dentate gyrus. **b1**. mCherry channel of b. **b2**. Dcx and DAPI channel of b. **b3**. BrdU channel of b. **c** Dcx, mCherry, and BrdU triple immunostaining with DAPI counterstain in Lv13-transduced APP/PS1dE9 mouse dentate gyrus. **c1.** mCherry channel of c. **c2.** Dcx and DAPI channel of c. **c3.** BrdU channel of c. **d** Dcx, mCherry, BrdU triple immunostaining with DAPI counterstain in Lv1613-transduced APP/PS1dE9 mouse dentate gyrus. **d1**. mCherry channel of d. **d2**. Dcx and DAPI channel of d. **d3**. BrdU channel of d. **e** Normalized values of Dcx-positive, BrdU-Dcx double positive and BrdU-Dcx-mCherry triple positive cells in wild type mouse dentate gyrus transduced with Lv13 (control) or Lv16 (NGFR). **f** Normalized values of Dcx-positive, BrdU-Dcx double positive and BrdU-Dcx-mCherry triple positive cells in APP/PS1dE9 mouse dentate gyrus transduced with Lv13 (control) or Lv16 (NGFR). Scale bars equal 50 μm. Error bars represent standard error of the means, with each point representing one section. Y-axes represent numbers per mm^2^. 3 mice were used.

**Fig. 4:**
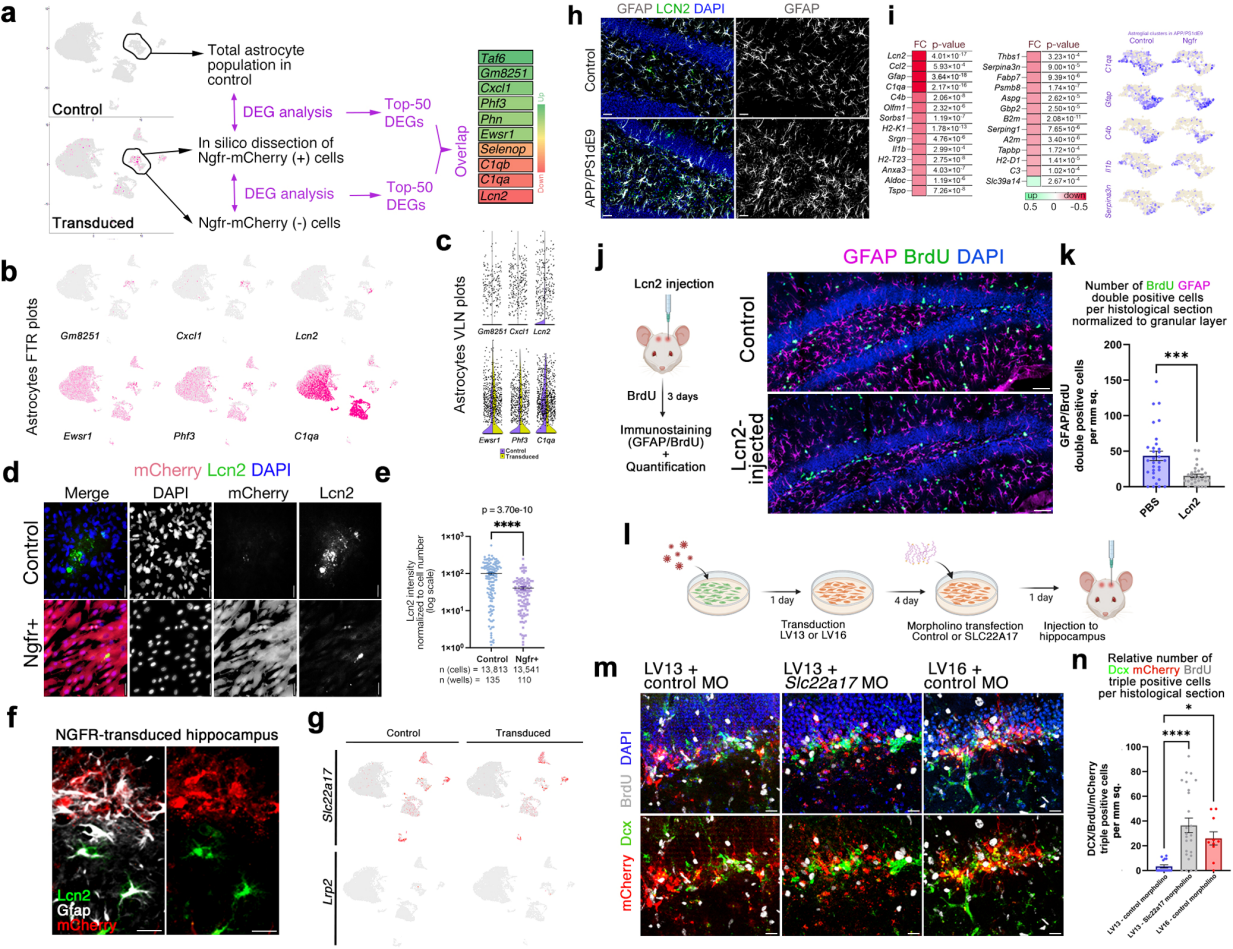
Ngfr regulates neurogenic response in DG astrocytes through suppression of Lcn2/Slc22a17 activity. **a** Single cell transcriptomics strategy for isolating Ngfr-mCherry positive astrocytes and comparing the transcriptomics profiles to non-transduced astrocytes or control astrocytes. The comparison showed differential expression of 10 genes in common as shown in the heat map. **b** Expression of selected genes on tSNE plots. **c** Violin plots for selected genes. **d** Immunostaining for Lcn2 and mCherry with DAPI counterstain in control and Ngfr+ mouse hippocampal astrocytes**. e** Quantification of Lcn2 signal intensity normalized to cell numbers. **f** Immunostaining for Lcn2, Gfap and mCherry in LV16 Ngfr-transduced DG. Lcn2-positive and mCherry positive astrocytes do not overlap. **g** tSNE plots for two receptors of Lcn2: *Lrp2, Slc22a17***. h** Gfap and Lcn2 immunostaining indicates increased Lcn2 and gliosis in APP/PS1dE9 mice. **i** Heatmap showing the changes in astrocyte A1 state markers upon Ngfr+ transduction. Astrocyte cluster 6 clusters showing expression of *C1qa, Gfap, C4b, Il1b*, and *Serpina3n* in control and Ngfr+ states. **j** Injection, BrdU treatment and analyses scheme of the effect of Lcn2 on proliferating GFAP cells, and immunohistochemical staining for BrdU, GFAP and DAPI. **k** Quantification graph for BrdU-GFAP double positive cells. **l** Schematic representation of the cell-type specific functional knockdown of *Slc22a17* in astrocytes. **m** mCherry, Dcx, BrdU immunostaining with DAPI counterstain in SGZs transplanted with Lv13+control morpholino, Lv13+*Slc22a17* morpholino and Lv16+control morpholino-treated astrocytes. Lower panels: DAPI omitted from upper panels **n** Quantification of Dcx, mCherry and BrdU triple positive cells in **i**. Slc22a17 knockdown mimics Ngfr transduction. *n* = 3. Scale bars equal 25 μm. Error bars represent standard error of the means.

To determine whether *Ngfr* expression could enhance proliferation and neurogenesis of the astroglia in DG, we performed Lv13 and Lv16 transduction, BrdU labeling, and quantified the extent of labeled astroglia (Gfap+) and newborn neurons (Dcx+) at 3 days after transduction in wild type and APP/PS1dE9 mouse model of AD (Figs. 2, 3 and Supplementary Fig. 5). We found that Ngfr expression enhanced proliferation and neurogenesis in both healthy (Fig. 2a–f and Supplementary Figs. 5, 3; Supplementary Data 4; by 2.99-fold and 2.08-fold, respectively) and AD mouse brains (Fig. 2g–l, Supplementary Fig. 5, and Fig. 3; Supplementary Data 4; by 4.85-fold and 7.32-fold), validating the Ngfr-mediated molecular changes in astroglia (Fig. 1). Ngfr expression also increased the total number of BrdU and mCherry double positive cells in both control and AD mice (Fig. 2m, n). To determine the effect of Ngfr on proliferation and neurogenesis in astrocytes, we studied the Ngfr-transduced astroglia in our single-cell sequencing by in silico dissection of Ngfr-mCherry expressing cells and determining the DEGs in comparison to non-transduced astroglia from the same mouse brain (analysis 1) or astroglia from control virus (Lv13) transduced mouse brain (analysis 2) (Fig. 4a and Supplementary Data 2, 3). We performed these analyses to ensure that Ngfr expression in astroglia is not a result of technical variables (by comparing the same astroglia population in the same animal according to their transduction status) or due to the presence of the lentiviral particles in the brain (comparing the astroglia from control Lv13 and Ngfr-containing Lv16-transduced animals).

### Ngfr signaling suppresses *Lcn2* expression in astroglia

When we selected the top-50 DEGs and selected the common hits in analyses 1 and 2, we found 10 genes that changed their expression upon NGFR expression in both analyses, confirming the robustness of our findings (Fig. 4a). We hypothesized that if a specific cell autonomous effect of Ngfr is running through a select number of genes in neurogenic astroglial population, then those genes could be mainly expressed in astroglia. Investigating the genes only expressed in astroglia would also minimize the effects of non-astroglial transduction of the viral particles. To determine whether any of the identified candidate genes has restricted astroglial expression, we generated tSNE plots and found that *Gm8251, Cxcl1*, and *Lcn2* were expressed predominantly in astroglia (Fig. 4b). By drawing violin plots, we observed that only *Lcn2* showed a significant change in expression in astroglia (Fig. 4c and Supplementary Fig. 4h). To validate our findings and to test whether Ngfr signaling has a cell autonomous effect on Lcn2 expression, we performed mono-cultures of mouse hippocampal astroglia in vitro and determined Lcn2 after transducing them with Lv13 and Lv16 viruses (Fig. 4d, e). We found that Ngfr can reduce Lcn2 protein levels in a cell autonomous manner in both control and amyloid-treated mouse astroglia, suggesting a cell autonomous effect (Fig. 4d, e). To confirm these findings in vivo, we performed immunohistochemical staining for Lcn2 in Lv16-transduced mouse DG and found that while non-transduced astroglia were Lcn2-positive, Ngfr-mCherry positive astroglia did not express Lcn2 (Fig. 4f), supporting the hypothesis that Ngfr signaling specifically reduces Lcn2 in DG astroglia.

### Ngfr signaling reduces reactive astroglial signatures

Lcn2 is a ligand and can bind to two receptors, Lrp2 and Slc22a17^35,36^. To determine whether these receptors are expressed in mouse DG, we generated two tSNE plots for the receptors and found that, while *Lrp2* is not expressed, *Slc22a17* is expressed in several cell types, including astroglia (Fig. 4e and Supplementary Fig. 4h), suggesting that Lcn2 can act via an auto/paracrine signaling as Lcn2 is upregulated in AD mice in astroglia concomitant to the reactive gliosis (Fig. 4h). We hypothesized that Ngfr expression in astroglia could therefore ameliorate the reactive gliotic A1 gene signatures^37–40^. We found that *Ngfr* expression in astroglia significantly reduced the expression levels A1 signature genes such as *Ccl2, Gfap, C1qa, C4b*, and *Il1b* in AD mouse brain (Fig. 4i and Supplementary Data 5, 6).

### Lcn2 acts via Slc22a17

Reactive state of glia reduces its proliferative and neurogenic ability^40,41^. To determine whether Lcn2 could alter the proliferation of astroglia, we injected Lcn2 into the hippocampus of the wild-type mice, treated the animals with BrdU and analyzed the labeled astroglia (Fig. 4j). We observed that Lcn2 significantly reduces the number of label-retaining Gfap+ cells, indicating reduced astroglial proliferation (Fig. 4k), which reminisce about the consequences of AD pathology on neurogenesis. To determine whether Lcn2-Slc22a17 signaling is biologically relevant to pro-neurogenic activity, we performed loss-of-function studies for *Slc22a17* (Fig. 4l–n) by using morpholino oligonucleotides that effectively reduce Slc22a17 protein levels (Supplementary Fig. 6). Since Slc22a17 is expressed in several cell types and to avoid pleiotropic effects of the loss-of-function that would confound the astroglia-related observations, we transduced the mouse DG astrocytes in vitro, treated the cells with control and *Slc22a17* morpholinos and then transplanted these astroglia to the SGZ of wild type mouse brains (Fig. 4l). With this method we generated a surrogate cell type-specific knockdown, as the blockage of Lcn2-Slc22a17 signaling in mCherry-positive astroglia could be achieved and assessed without altering this signaling pathway in other cell types. To determine the progeny of the transplanted astroglia, we injected the mice with BrdU after transplantation. After performing immunohistochemical staining for BrdU (newborn cells), Dcx (early neurons) and mCherry (transduced cells), we found that compared to Lv13 and control morpholino, Lv13 and *Slc22a17* morpholino significantly increased the generation of newborn neurons from transplanted astrocytes and this increase is comparable to Lv16 (Ngfr) transduction alone (Fig. 4m, n and Supplementary Data 4). These results suggest that the imposition of pro-neurogenic potential by Ngfr expression to DG astroglia might be cell autonomous through suppressed autocrine Lcn2-Slc22a17 signaling, which favors non-neurogenic reactive astroglial state.

### Ngfr signaling and induced neurogenesis reduces amyloid load and Tau hyperphosphorylation

**Fig. 5:**
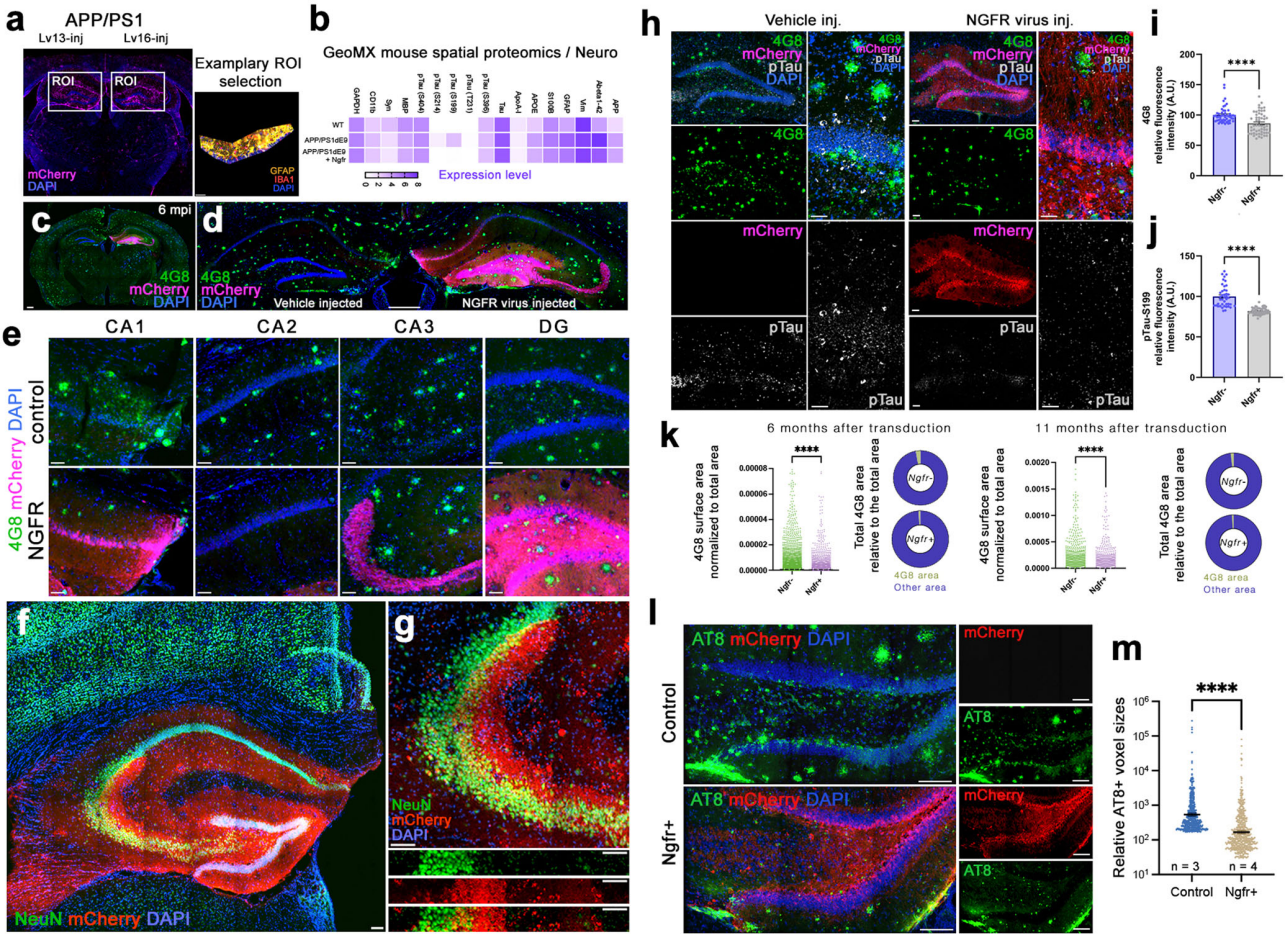
Ngfr reduces Aβ42 load and phosphorylated Tau in the hippocampus of APP/PS1dE9 mice. **a** Representative image from GeoMx spatial proteomics mouse brain section and region of interest (ROI) at the SGZ. **b** Heatmap showing the detection levels of selected proteins after *Ngfr* transduction in wild type and APP/PS1dE9 mice. **c** Representative APP/PS1dE9 mouse brain section immunostained for 4G8 and mCherry with DAPI counterstain at 6 months after transduction with Lv16 in one DG. **d** High-magnification image from **c**. **e** Comparison of amyloid load in CA1, CA2, CA3 and DG regions of control and *Ngfr*-transduced hemispheres. **f** Double immunostaining for NeuN and mCherry with DAPI counterstain at 6 months after transduction. **g** Higher magnification image of **f**. Individual channel panels indicate overlapping mCherry and NeuN. **h** Quantification of amyloid load in terms of normalized amyloid immunoreactivity by comparing control (v) versus *Ngfr* (Lv16) transduction. *n* = 5. **i**. 4G8, mCherry, phosphorylated Tau-S199 immunostaining with DAPI counterstain in control and *Ngfr*-injected DGs. Individual fluorescence channels and close-up images are also shown. **j** Quantification of pTau-S199 in terms of normalized fluorescence by comparing control (v) versus *Ngfr* (Lv16) transduction. *n* = 5. **k** 4G8 surface area quantification and relative 4G8 area graphs for 6 months and 11 months after Ngfr transduction. **l** Double immunolabeling for AT8 and mCherry with DAPI counterstain in control and Ngfr+ animals. Individual panels show single fluorescent channels. **m** Quantification of relative AT8 immunoreactivity. Ngfr reduces the prevalence of AT8. Error bars represent standard error of the means. Scale bars equal 100 μm.

Since Ngfr signaling in DG astroglia induced proliferation and neurogenesis, and suppressed the molecular pathways related to AD (Figs. 1–4), we hypothesized that active Ngfr signaling might alter the build-up of the AD pathology^14,42–45^. To determine whether Ngfr signaling would alter AD-related proteins that cannot be identified by transcriptomics, we performed spatial proteomics on mCherry-enriched regions of Lv13 and Lv16-transduced SGZs in wild type and APP/PS1dE9 AD model (Fig. 5a). After data normalization and quality control (Supplementary Fig. 7 and Supplementary Data 7, 8), we plotted the fold changes in protein expression levels between Ngfr and control transduction (Fig. 5b for selected proteins, Supplementary Fig. 8 for entire panel). We found that among the highest fold changes upon Ngfr transduction in APP/PS1dE9 mouse were for Aβ42 (−47%), phosphorylated Tau (S199) (−75%) and phosphorylated Tau (S214) (−42%) (Fig. 5b). Other AD-related proteins such as pTau-S396, pTau-S404, and APOE were also downregulated by NGFR (Fig. 5b, Supplementary Data 8), suggesting that activation of Ngfr signaling in DG and particularly in astroglia could improve AD pathology burden. To test this hypothesis, we transduced the APP/PS1dE9 mice with Lv16 and analyzed these brains 6 months after transduction (Fig. 5c). mCherry signal in the transduced hemisphere covered the entire DG (Fig. 5d). Our injection paradigm transduces approximately 100 astroglia per brain and obtaining thousands of distantly migrated mCherry-positive cells at 6 months after transduction indicates that Ngfr-transduced astrocytes significantly contributed to neurogenesis (Fig. 5d, e). To test this, we performed double immunolabeling for NeuN (neurons) and mCherry (transduced cells), and found that transduced cells overlap with hippocampal neurons (Fig. 5f, g). When different subregions of the hippocampus were analyzed, we observed reduced immunoreactivity for 4G8-positive amyloid plaques (Fig. 5h), and this reduction amounted to 22% in the entire hippocampus (Fig. 5h, i). Since spatial proteomics showed reduced levels of phosphorylated Tau (Fig. 5b), we tested these findings by performing immunohistochemical staining for pTau-S199 in control and Ngfr-transduced hemispheres of APP/PS1dE9 mouse DG (Fig. 5h). We observed that Ngfr-transduced DGs have significantly lower amounts of pTau-S199 in the overall hippocampus (11%, Fig. 5h, j), while the difference in SGZ is more pronounced (Fig. 5h). We confirmed these findings at a later time point at 11 months after transduction (Supplementary Fig. 9). In addition to the fluorescent levels, we also quantified the surface area of amyloid plaques normalized to the total hippocampal area of analyses (Fig. 5k). In both 6 months and 11 months after the transduction, Ngfr+ hippocampi significantly reduced the 4G8-positive plaque surface (-%66,3 at 6 months and −48,1% at 11 months. At 6 months: in 6 Ngfr-, 5 Ngfr+ animals, 91,015 4G8-positive objects were analyzed in 60 brains sections; at 11 months: in 4 Ngfr-, 5 Ngfr+ animals, 51,583 4G8-positive objects were analyzed in 26 brain sections) (Fig. 5k and Supplementary Data 4). More advanced Tau phosphorylation patterns such as AT8-immunoreactivity were also reduced by Ngfr expression at 6 months after transduction (Fig. 5l, m). In overall, these results suggest that Ngfr signaling reduces amyloid and Tau pathology concomitant to enhancing adult hippocampal neurogenesis in APP/PS1dE9 mouse model of Alzheimer’s disease.

### NGFR signaling overlaps with proliferative markers in human brain and reduces with aging

Since Ngfr signaling can be functionally associated with neurogenic ability of astroglia, we hypothesized that through earlier stages of brain development in humans, when extensive neurogenesis takes place, *Ngfr* expression might be observed, and this prevalence could reduce with aging. To determine whether *Ngfr* expression through any period of the human brain development and maturation would overlap with proliferating astroglia, we analyzed three publicly available single-cell sequencing datasets (human brain organoids, fetal human brain, adult human brain) (Supplementary Fig. 10)^46,47^. Cell clustering, marker gene identification, and cell type determination followed by generation of tSNE plots for *NGFR* and *PCNA* (proliferating cell marker) showed that in brain organoids and developing fetal brain, *NGFR* expression partially overlaps to proliferating astroglial cells while in adult human brains, *NGFR* is not expressed in astroglia (Supplementary Fig. 10), suggesting an age-related loss of *NGFR* activity that might correlate with reduced neurogenesis.

### LCN2 increases in human brains with Alzheimer’s disease and correlates with the amyloid load

**Fig. 6:**
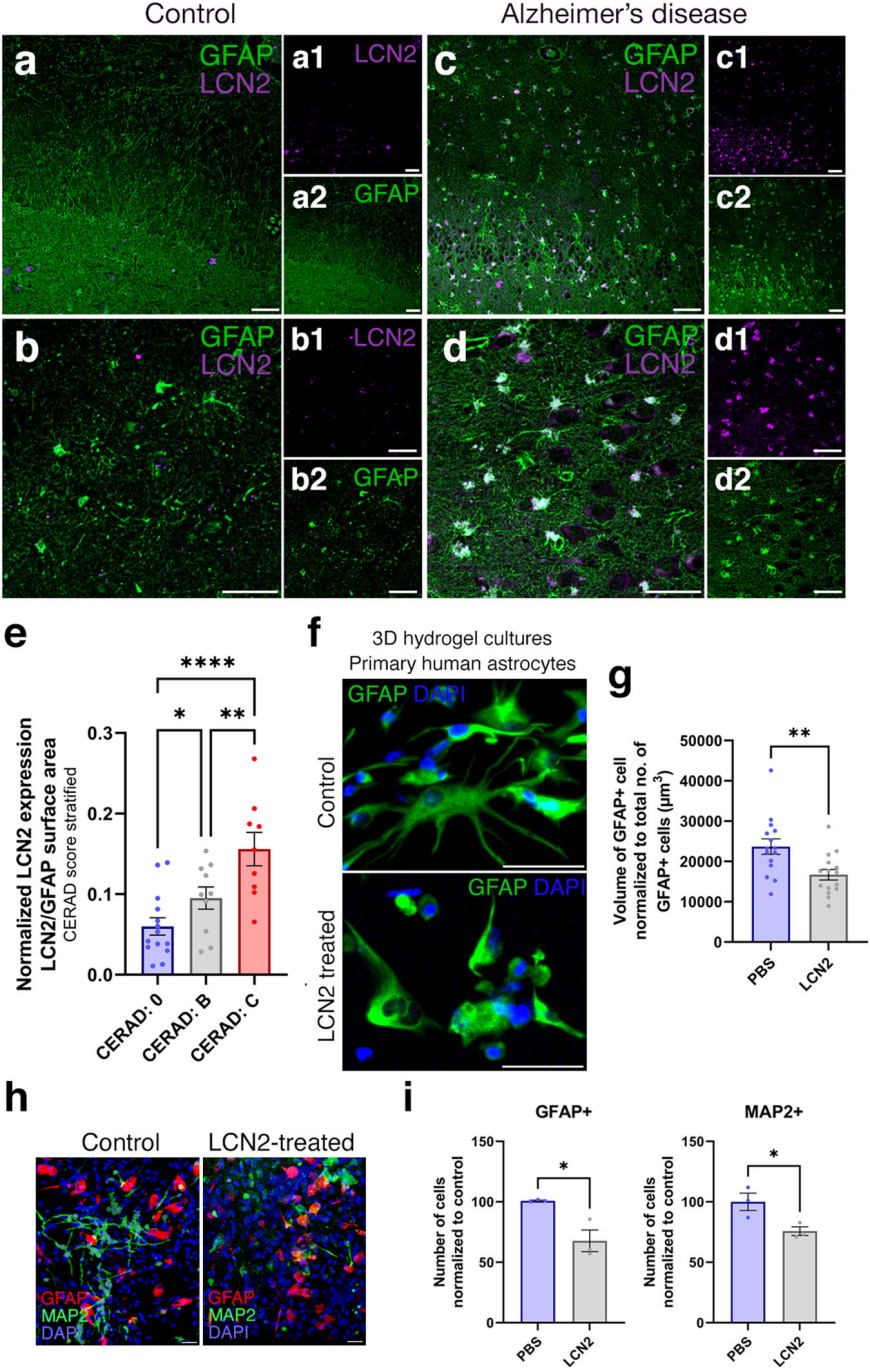
LCN2 is upregulated in human brains with AD. Immunohistochemical stainings for LCN2 and GFAP on hippocampal brain sections of healthy control (**a**, **b**) and CERAD score C AD patient (**c**, **d**). Black-white insets (**a1**–**d2**) indicate individual fluorescent channels. LCN2 (top) and GFAP (bottom). **e** Quantification of LCN2-positive GFAP cells normalized to total GFAP cells. In total *n* = 7 human brains, *n* = 21 images analyzed. ***p < 0.001. **f** Immunostaining for GFAP on primary human astrocytes in 3D hydrogels: control and LCN2-treated. **g** Quantification of the volume of GFAP normalized to total number of GFAP cells. LCN2-treatment reduces the volume of astroglia, indicative or reactive states. Scale bars equal 50 μm. **h** GFAP and MAP2 immunostaining on control and LCN2-treated 3D hydrogel cultures of human primary fetal astroglia. **i** Quantification of normalized GFAP and MAP2-positive cells after the culture period. Scale bars equal 25 μm. Error bars represent standard error of the means.

We hypothesized that the expression of *LCN2* could increase in the brains of AD patients, where neurogenesis is reduced. To test this hypothesis and to correlate our findings to human brains, we performed immunolabeling of human hippocampi for GFAP and LCN2 in healthy controls and AD patients (Fig. 6 and Supplementary Table 2). We observed scarce LCN2 expression in healthy aged individuals (Fig. 6a, b), while in human AD brains LCN2-positive astroglia increases dramatically concomitant to architectural changes of glial extensions and elevated hypertrophy (Fig. 6c, d). To determine whether LCN2 expression levels correlate with neurotic plaques, we stratified the individuals according to their CERAD scores (Fig. 6e). Our quantifications of LCN2-positive GFAP+ cells normalized to the overall GFAP+ cells indicated that the number of LCN2-positive glia increases significantly with advancing CERAD scores, suggesting that LCN2 expression in human brains correlate with increased number or neuritic amyloid plaques (Fig. 6e).

### LCN2 is sufficient to reduce neurogenesis in human astroglia

In human brains, we also observed that the LCN2-expressing astroglia were more compact than their healthy counterparts (Fig. 6b, d). Therefore, to determine whether LCN2 is sufficient to alter the morphology of astroglia, we used 3D starPEG-Heparin hydrogel cultures of primary human astroglia (Fig. 6f). We observed that treatment of astroglia with LCN2 significantly reduces the glial volumes indicative of a reactive state (Fig. 6g), consistent with the reduction of GFAP and MAP2-positive cells, indicative of gliosis-dependent reduction of proliferation and pro-neurogenic potential (Fig. 6h, i). Since reactive astrocytes can both increase their proliferation at the boundaries of tissue damage or toxicity while reduce their proliferation at other regions^48^, we hypothesize that since the 3D cultures we used do not have plaques or tissue damage, the reduced proliferative response might resemble the parenchymal reactive astrocyte phenotypes when considered together with the reduced neurogenic outcomes.

### Potential downstream regulators of NGFR signaling in humans, mouse and zebrafish

**Fig. 7:**
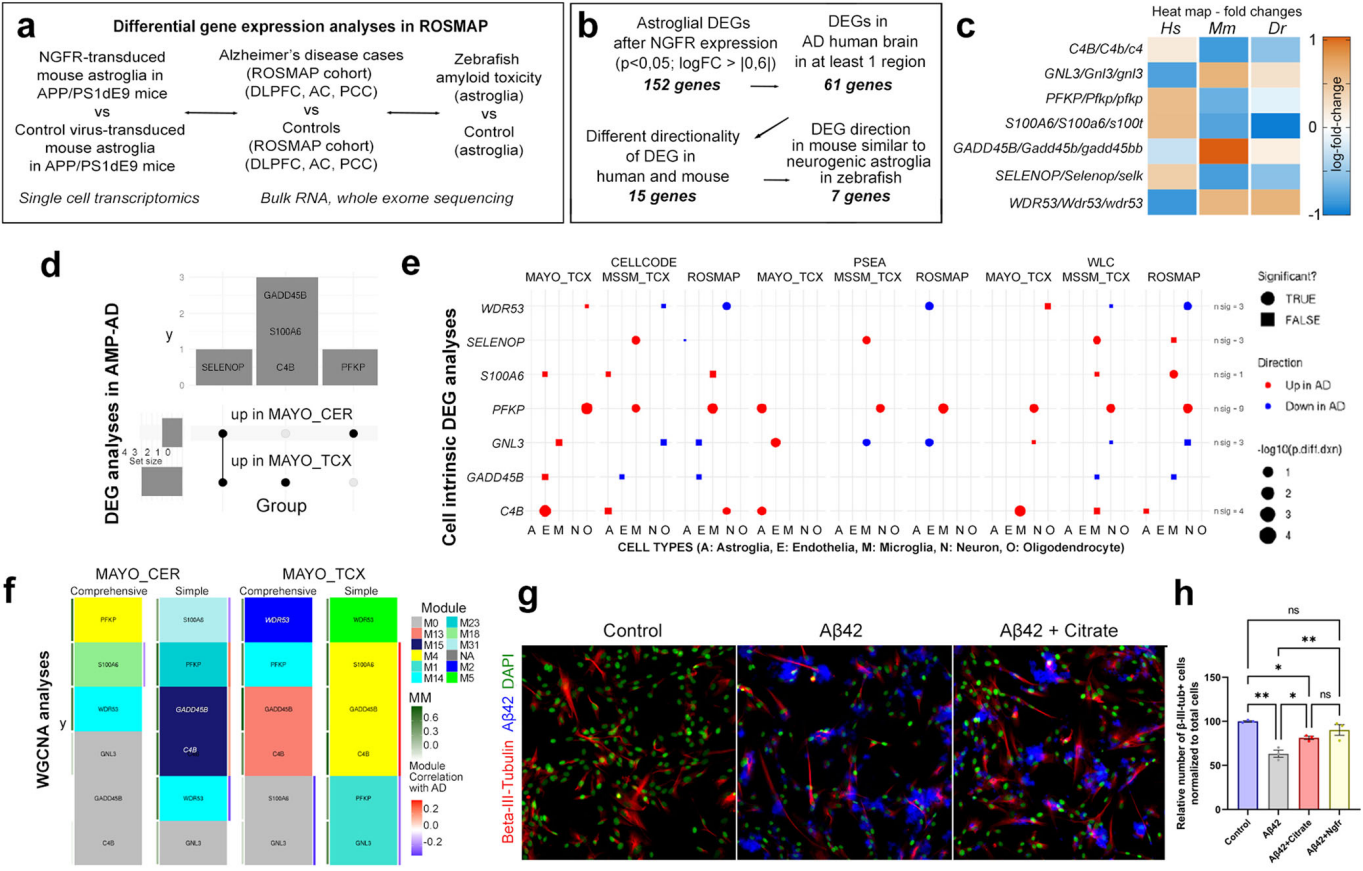
Comparison of gene expression changes in mouse brain with NGFR to human AD cohorts by differential gene expression analyses, cell intrinsic gene expression and Weighted gene co-expression network analysis. **a** Schematic flow of the differential gene expression analyses with ROSMAP AD cohort. **b** Stringency criteria and number of genes in each comparison category. **c** Heat map of expression changes of 7 candidate genes. Hs: human, Mm: mouse, Dr: zebrafish. **d** Overlap of significant DEG (FDR < 0.05 in the AMP-AD datasets. **e** Cell-intrinsic DEGs in 5 different cell types calculated using 3 different analytic tools (CellCODE, PSEA, WLC) from 3 different datasets (Mayo, MSSM, ROSMAP). Color indicates direction of changes. Circles are significant changes (*p* < 0.05) while squares are not. **f** The gene of interest and their assigned modules (tile color) in WGCNA networks constructed from Mayo CER and TCX datasets either adjusting (comprehensive) or not adjusted (simple) for the cell proportion changes. Red-Blue color tile to the right indicates module correlation to AD diagnosis, where only significant correlation (*p* < 0.05) is shown. Green tile to the left indicates the gene’s module membership with respect to its assigned module. **g** Beta-III-tubulin and Aβ42 immunocytochemical staining with DAPI counterstain on control, Aβ42-treated and Aβ42+citrate-treated primary human astrocytes. **h**. Quantification of neurons in conditions in g and Aβ42-treated Ngfr-transduced primary human astrocytes. Scale bars equal 25 μm. Error bars represent standard error of the means.

To further investigate the role of NGFR signaling in AD, we compared the transcriptional changes exerted by *Ngfr* expression in DG of APP/PS1dE9 mouse (Data S6) to large AD human cohorts in humans (Fig. 7). We used the ROSMAP study and its bulk RNA sequencing from the dorsolateral prefrontal cortex (DLPFC), anterior caudate (AC) and posterior cingulate cortex (PCC) regions^49,50^ (Fig. 7a). To add another stringency level and a biologically relevant experimental animal model, we also included data from sorted astroglia of an amyloid toxicity model of adult zebrafish brain, where the induced pathology elicits an Ngfr-dependent neurogenic response^19,23^ (Fig. 7a). In mouse astroglia transduced with *Ngfr* versus control astroglia, we identified 152 genes that are significantly DEGs, of which 61 are also nominally significant DEG in at least one ROSMAP brain region (Fig. 7b and Supplementary Data S). We hypothesized that the pathology-altering and neurogenic effects of Ngfr activity could be reflected in the gene expression patterns of these overlapping 61 genes. Hypothetically, in mouse and human AD brains, the genes must have opposite directionality of change of expression. We found 15 genes conform to this criterion (Fig. 7b and Supplementary Data 6). Since zebrafish astroglia maintains pro-neurogenic states after amyloid toxicity upon *Ngfr* signaling, we used zebrafish brain as a reference comparison and hypothesized that the expression change in those genes should be same direction with mouse after Ngfr transduction but opposite direction with the human AD cohorts. 7 genes conformed to these criteria (Fig. 7b, c). Their expression heatmap indicated that in human AD brains, *WDR53, GADD45B, and GLN3* are downregulated while *C4B, PFKP, S100A6,* and *SELENOP* are upregulated (Fig. 7c), while *Ngfr* transduction in mouse hippocampus or amyloid toxicity in adult zebrafish brain changed these genes in opposite directionality (Fig. 7c).

### Weighed co-expression and deconvoluted bulk RNA sequencing analyses show the relevance of NGFR to pathological gene expression modules in human Alzheimer’s disease brains

To further analyze the involvement of these 7 genes in AD brains, we assessed their expression by three different approaches. (1) At the bulk tissue RNA expression level, we collected DEGs comparing AD and control brains from the AMP-AD datasets representing seven distinct datasets^49,51–53^ (Fig. 7d). (2) For bulk analyses, only those genes differentially expressed at FDR < 0.05 were considered DEGs. At the cell type level, we retrieved the cell-intrinsic DEGs (CI-DEGs) comparing gene expression levels between AD and control brains obtained using deconvoluted bulk gene expression from three independent bulk gene expression datasets via three different methods as previously described^54^ (Fig. 7e). (3) At the gene network level, we retrieved the information about the co-expression modules^55^ where the gene of interest is a member of the network module. The modules were constructed using AMP-AD Mayo Clinic temporal cortex (Mayo TCX) and cerebellum (CER) datasets as previously described^56,57^ (Fig. 7f). Two analytic models were applied, a comprehensive model that adjusts for cell type markers to account for cell population variation in bulk tissue, and a simple model that does not. Relevant network information includes the module membership, module correlation with AD diagnosis, as well as enriched GO terms in the modules (Fig. 7d–f and Supplementary Data 10). Five of the 7 genes are DEGs (FDR < 0.05) in the Mayo Clinic AD cohorts analyzed, although nominally significant DEGs were observed in other cohorts for these and other genes (Supplementary Data 10). *SELENOP* is up in both Mayo CER and TCX, *C4B*, *S100A6*, and *GADD45B* are up in Mayo TCX, while *PFKP* is up in Mayo CER (Fig. 7d, Supplementary Data 10). In the CI-DEG datasets, 6 out of the 7 genes were significantly (*p* < 0.05) differentially expressed in at least one of the cell types in any of the three brain regions (Fig. 7e and Supplementary Data 10). *PFKP* showed consistent upregulation in AD. Its upregulation is detected in neurons in 4/9 analyses, microglia (*N* = 3), astrocytes (*N* = 1), and oligodendrocytes (*N* = 1), but not in endothelia. Another gene that has CI-DEGs in astrocytes (*N* = 3) is *C4B*, which is also up-regulated in endothelia (*N* = 1), microglia (*N* = 2), and neurons (*N* = 1). These findings provide further support for human brain expression changes in *C4B, PFKP, S100A6* and *SELENOP* that are biologically congruent with those from mouse and zebrafish models. Of these *C4B* and *PFKP* are also consistent CI-DEGs in neurons and glia. Of the 4 types of co-expression modules evaluated (2 brain regions, 2 analytic models), *C4B* resides within Mayo TCX Simple Module M4, TCX Comprehensive M13, and CER Simple M15 (Fig. 7f, Supplementary Data 10). These modules are enriched in microglia and endothelia genes, where *C4B* is a highly connected hub gene in the TCX Simple M4 (module membership = MM = 0.8). Further, both CER Simple M15 and TCX Simple M4 have positive correlations with AD diagnosis, consistent with the DEG and CI-DEG results. Interestingly, *GADD45B* resides in the same modules as *C4B*, suggesting co-regulation of these genes. *PFKP*, one subunit of Phosphofructokinase 1^58^, is a member of the TCX Simple M1, TCX Comprehensive M14, and CER Comprehensive M4 modules, all of which are enriched for neuronal genes, in addition to CER Simple M23. *PFKP* is a hub gene for both CERs but not for the TCX modules. Both Simple modules CER M23 and TCX M1 are correlated with AD, former showing higher levels, consistent with DEG results, though latter showing lower levels, likely driven by other genes in this neuronal module enriched for synaptic biological terms.

### Phosphofructokinase (*PFKP*) is co-expressed with *NGFR* in human Alzheimer’s disease brains and allosteric blockage of PFKP enhances neurogenesis in human astroglia

To determine whether modulation of function of *NGFR* co-expressed gene module member *PFKP* would mimic the enhanced neurogenic outcome in human astrocytes, we used primary human cortical astrocytes in vitro and blocked PFK function by using its allosteric regulator citrate^59^. We found that antagonizing PFK ameliorated Aβ42-induced reduction in formation of neurons (β-III-Tubulin+, Fig. 7g) comparable to Ngfr+ expression in primary human astrocyte cultures in vitro (Fig. 7h). Taken together, the human brain gene expression data support robust expression changes in AD brains in a direction congruent with the cross-species data, and evidence of neuronal and glial expression perturbations for *PFKP*, which is co-expressed with other microglial/endothelial and neuronal genes, respectively, and can mimic NGFR-induced neurogenic plasticity in astroglia.

## Discussion

**Fig. 8:**
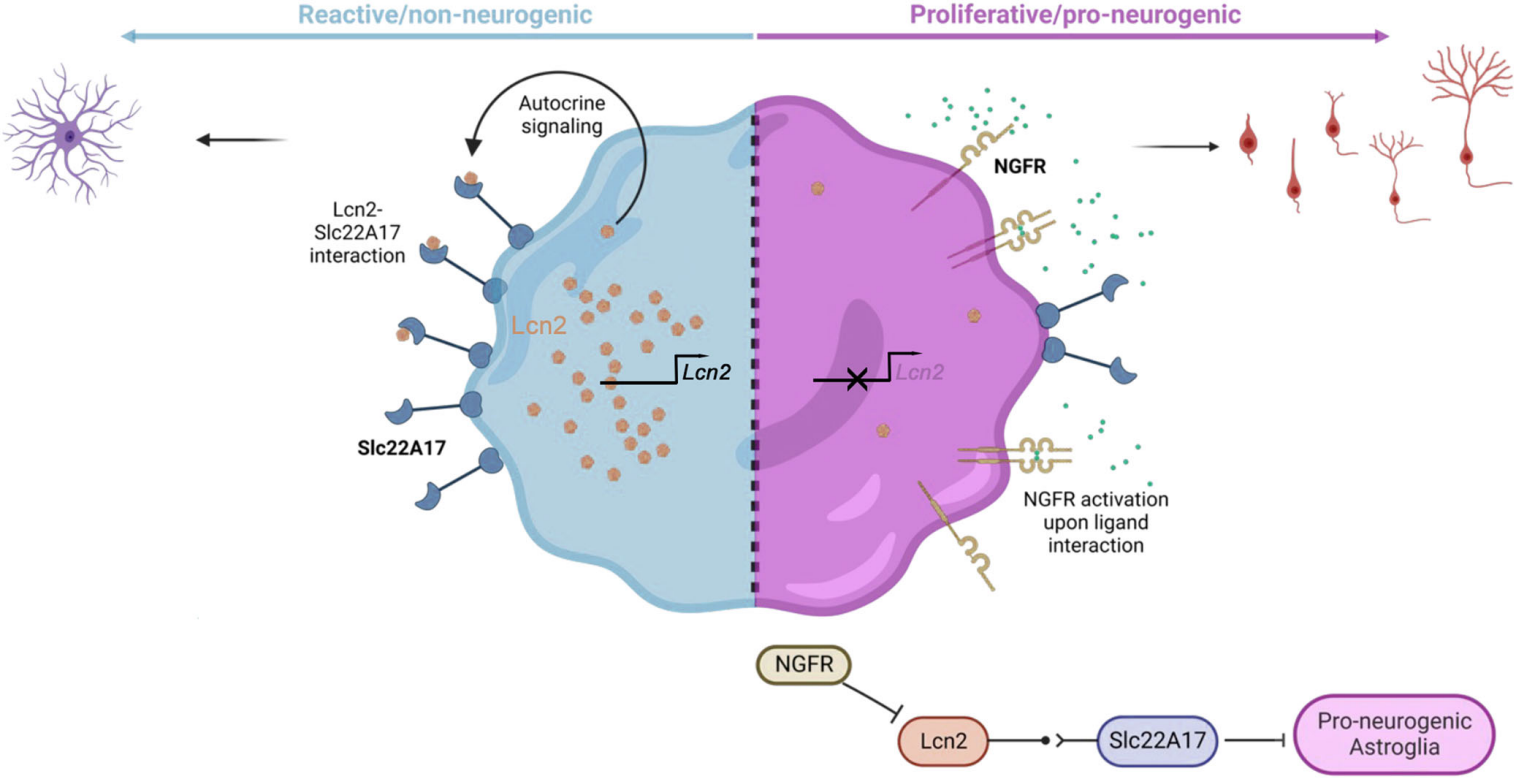
Schematic view of the NGFR/LCN2/SLC22A17-dependent neurogenic switch mechanism in astroglia. Nerve growth factor receptor can convert reactive astroglia to neurogenic state in mouse model of Alzheimer’s disease (AD) through suppression of Lcn2 activity on Slc22a17 receptor. In AD cohorts in humans, *NGFR* is co-expressed with a weighed expression cluster including *PFKP*, blockage of which enhances neurogenesis from astroglia. In zebrafish, Ngfr activity determines the neuroregenerative potential after amyloid toxicity. We propose an evolutionarily conserved mechanism relating to neurogenesis-related brain resilience in vertebrates.

In this study, we discovered that an autocrine molecular mechanism - Lipocalin-2 (Lcn2) / Solute carrier protein 22a17 (Slc22a17) axis - regulates the neurogenic potential of astroglia by controlling the pro-neurogenic versus reactive states (Fig. 8). Ngfr-induced neurogenesis is concomitant to reduced amyloid pathology and Tau phosphorylation in mice. We found that in humans, LCN2 is elevated in AD patient brains parallel to the prevalence of the neuritic plaques, suggesting a relationship between reduced neurogenic capacity and the etiology of these diseases, which needs to be further addressed. By using the single cell sequencing datasets, we also show that in fetal human brains, 3D brain organoids and human AD patient brains, *NGFR* expression defines neurogenic capacity that declines with age. Comparison of our findings to human transcriptomics datasets in AD cohorts indicated candidate genes that might play a role in neurogenic switch mechanism in mammalian astroglia. We also propose that evolutionary determinants of pro-neurogenic and neuro-regenerative potential can be identified by cross-species comparison to zebrafish, and engineered induction of regenerative programs could help design novel therapeutic routes in AD in humans. Our study identified Slc22a17 as a potential drug target to design therapies for interventions to enhance neurogenesis in Alzheimer’s disease and proposed additional candidate genes potentially functioning in the NGFR/p75NTR pathway. One such gene is *PFKP*, we found to be a negative regulator of neurogenesis.

The reduced neurogenesis outcome in Alzheimer’s disease could be a pathological culprit of the disease^14,15,42,60^. Therefore, nudging the astroglia - the endogenous reservoir of cells that bear neurogenic potential - to favor neurogenesis instead of reactive states during the course of the neurodegenerative disease pathology could help to restore the brain’s resilience and ability to cope with neurodegeneration^43,45^. However, the understanding of the molecular mechanisms how such a neurogenic competency can be imposed to switch astroglia is insufficient. Previous studies showed that neurotrophin signaling, especially BDNF, imposes a neuroprotective ability in neurons^61,62^ and can contribute to the amelioration of cognitive decline in AD^44,63^. Major neurotrophin receptor TrkB was associated with neuronal survival; however, BDNF is not directly inducing neural progenitor proliferation and neurogenesis in rodent brains^44^. We previously showed that unlike mammals, an experimental adult zebrafish AD brain model uses BDNF to impose and maintain the pro-neurogenic ability of astroglia through direct induction of proliferation and neurogenesis^19^. We found that this activity was dependent on Ngfr/p75Ntr receptor but not TrkB. NGFR can be bound by various ligands such as BDNF or NGF or their pro forms^64^. In the hippocampus, we did not detect active cleaved forms of Bdnf or Ngf in the SGZ, but pro-Bdnf is expressed in this region (Fig. S10). This is consistent with the binding ability of pro-Bdnf to Ngfr^65^, and suggests that Ngfr/pro-Bdnf signaling could be responsible for neurogenic conversion of mouse astroglia. Therefore, here we show that transferring the molecular understanding from organisms with neuroregenerative ability, such as zebrafish, to mammalian brains is a promising cross-species approach to study the neuroregenerative ability to counteract AD pathology through fate-switching of the astroglial cell populations that bear neurogenic potential but cannot manifest this ability under the pathological conditions that favor reactive state.

We found that *Lcn2* expression is negatively regulated by Ngfr signaling in mouse astroglia, and *Lcn2* and its receptor *Slc22a17* negatively regulates astrocyte proliferation and neurogenesis. This ultimately means that neurogenesis can be modified by targeting specific signaling pathways and the resilience of the brain in disease can be modified through controlling the physiological state of astroglia. Lcn2 is a secreted protein that is associated with autocrine or paracrine induction of reactive astrocyte state and induce hypertrophic Gfap expression^66^. *Lcn2* is upregulated in several brain diseases and may contribute to neurodegeneration, promote cell death and inflammation^67^. In this study, we found that LCN2 is also upregulated in human AD, consistent with previous findings^68^. However, the link between Ngfr signaling in astrocytes and Lcn2 has not yet been documented. Here, we propose that Ngfr signaling is sufficient to downregulate Lcn2 and promote pro-neurogenic state by suppressing the molecular signatures of reactive astrogliosis under AD pathology. Our spatial proteomics and single-cell transcriptomics data support this hypothesis (Fig. 4, Supplementary Figs. 7, 8, 12 and Supplementary Data 6). Additionally, our spatial proteomics analyses showed that, among the immune-related proteins, Cd44 was the most downregulated gene after Ngfr expression (Supplementary Fig. 8), which is consistent with previous independent findings that LCN2 promoted CD44^69^, an important cell surface molecule involved in regulating inflammation-dependent cellular response^70^.

We propose that NGFR signaling is an evolutionary determinant of the neurogenic potential in astroglia. In zebrafish, Ngfr signaling is naturally active in astroglia with neurogenic potential throughout the lifespan, which correlates with the life-long proliferation and neurogenic activity^19^, while in the mouse brain, Ngfr is not present in hippocampal astroglia. When Ngfr is activated, reactive astrocyte programs are hampered through suppression of Lcn2 and pro-neurogenic programs prevail. Therefore, we hypothesize that mammalian brains could have lost NGFR expression in adult brain astroglia through the evolution, and this could have disabled the switches from reactive neurogenic state to the pro-neurogenic state in diseased mammalian brains. This hypothesis is supported by findings that a small population of astrocytes (0.3%) in the subventricular zone (SVZ) of mouse astrocytes are Ngfr-positive and they constitute a highly proliferative and pro-neurogenic subset of astroglia^71^, and Ngfr signaling could regulate genes related to cell proliferation, differentiation and neurite growth in vitro^72^. Our findings on single-cell sequencing data of human brain organoids, fetal human brain, and adult human brains (Supplementary Fig. 9) also confirmed that *NGFR* expression in astroglia declines with age and could contribute to the hampered neurogenic outcome or inability of adult human brains to fulfill efficient neuro-regeneration. In AD patients, LCN2-positive reactive astroglia increase in correlation with the prevalence of neuritic plaques (Fig. 6), suggesting that NGFR/LCN2/SLC22A17 signaling axis could be a critical fate determination step between neurogenic versus reactive gliotic response in disease. We also observed a reduction of pTAU-S199, a critical residue that is phosphorylated by CDK5 at late stages before the neurofibrillary tangle formation and is one of the residues detected by AT8 immunostaining^73^, after Ngfr transduction (Fig. 5). Therefore, the reduction in Aβ42 and phosphorylated forms of Tau protein could explain the long-term benefit of active NGFR signaling in astroglia.

The development of the Alzheimer’s disease pathology is a complex and multifaceted process, the genetic basis of which is still not fully elucidated^27,74–77^. Based on our data, we hypothesize that NGFR could regulate LCN2 expression through a potential signal cascade including NFkB. By using an integrated database for experimentally-verified transcriptional factor binding sites in mouse genome^78^ and comparing these transcriptional factors in our differentially expressed genes upon NGFR expression in astrocytes (Supplementary Data 6), we found that the expression levels of two transcriptional factors – *Stat1* and *Irf8* – were downregulated after *Ngfr* expression (log fold changes: −0,27 and −0,38, respectively). Stat1 was shown to regulate Irf8 expression to mediate interferon signaling^79–81^ and these two factors enhance the promoter occupancy of the interferon target genes in an inflammation-dependent manner^82^. Irf8 and Stat1 are critical for transforming glia into reactive phenotype^83^ and required for induction of Lcn2 expression^84,85^. One of the critical regulators of Stat1 and reactive phenotypes is NFkB signal transduction^86–88^. Our single cell sequencing datasets showed that Ngfr expression in astrocytes increase the expression of *Nfkbia* (log fold change 0,37, Supplementary Data 6), an NFkB inhibitor^89,90^. Previous studies also showed direct regulation of Lcn2 expression by NFkB^91,92^. Therefore, we speculate that Ngfr signaling in astroglia could suppress *Lcn2* expression through regulation of interferon signaling via downregulation of critical transcriptional mediators *Stat1* and *Irf8*, and through upregulation of *Nfkbia* that hampers the NFkB signaling. In support of this hypothesis, we found that the pathway and GO-term analyses on the Ngfr-dependent differentially expressed genes in astroglia highly enriched interferon signaling-related pathways (Supplementary Fig. 12a). Further experiments on specific temporal regulation of interferon pathways and detailed functional investigation of potential candidate regulators in astroglia in relation to Ngfr activity is warranted for detailed epistatic and biochemical interactions regulating the neurogenic potential of astroglia.

In our study, we hypothesized that Ngfr-mediated suppression of Lcn2 signaling would transform astroglia from a reactive phase to neurogenic or progenitor phase. To validate our findings, we compared the gene expression changes we observed to a longitudinal single cell transcriptome atlas of the human hippocampus^93^. We observed that two astrocyte populations that define age-dependent reactive (AST1, human reactive astrocytes that increase with age^93^) and progenitor (AST2, human progenitor astrocytes that reduce with age^93^) physiology paralleled well with the directions of gene expression changes Ngfr dictates in mouse astroglia (Supplementary Fig. 12b). For instance, Ngfr reduces AST1 markers *Gfap, Aqp4, Vim, C3, Dbi*, while increases AST2 markers *Ttc28, Usp24, Akap12, Egfr, Tmem131* and *Apc* (Supplementary Fig. 11b). These results cross validate our findings in mouse in humans, and when combined with our findings that Ngfr signaling may correlate with the age-dependent neurogenic and proliferative capacity of astroglia (Supplementary Fig. 10) suggest an evolutionary relevance of Ngfr signaling to astroglial potential for neurogenesis. Furthermore, besides the cell autonomous role of Ngfr in astroglia, we determined that in other cell types, non-cell autonomous changes could also take place. Our single cell transcriptomics data comparing Ngfr-transduced brains versus controls showed that in neuronal, microglial and oligodendrocyte cell populations, molecular pathways related to unfolded protein response, clearance of misfolded proteins, protein processing, proteasome and Tau protein binding were enriched (Supplementary Fig. 12c). This suggests that NGFR signaling may induce more efficient clearance of toxic proteins, which might explain the long-term reduction of amyloid and Tau burden in Ngfr-transduced mouse brains (Fig. 5). This is consistent with independent previous findings^94^.

We identified a small set of genes that might be regulated by NGFR signaling in vertebrate brains (Fig. 7 and Supplementary Data. 9, 10). Particularly, *PFKP* and *C4B* are upregulated in human AD cohorts (Fig. 7) but downregulated with *Ngfr* transduction in astroglia as well as upon amyloid toxicity in adult zebrafish brain (Fig. 7). *C4B* is a complement protein that is involved in classical activation pathway^95^, and found in AD patients cerebrospinal fluid as a biomarker for disease progression^96^. Our analyses found *C4B* in human brains upregulated in bulk tissue, astrocytes and other glia and its expression correlates with the expression modules enriched for microglial/endothelial genes (Fig. 7 and Supplementary Data 10). This suggests that the expression of *C4B* in astroglia could have an immunomodulatory effect regulated by NGFR signaling or counteracting the reactive gliotic state with NGFR could have ramifications in reducing the neuroinflammatory environment. This hypothesis is reasonable, as astroglia can modulate the immune environment and the progression of disease pathology^28,97–99^. *PFKP* is an enzyme subunit that regulates the glycolytic pathway. We found this gene consistently upregulated in human AD brains (Fig. 7 and Supplementary Data 9, 10) and residing in neuronally enriched expression modules correlated with AD (Fig. 7f and Supplementary Data 10). Alteration of energy metabolism in AD is a prominent pathological mechanism^100,101^ and regulation of *PFKP* by NGFR signaling could be a mechanistic link to AD neuropathology. Our findings that allosteric antagonism of PFKP leads to increased neurogenesis in primary human astrocytes (Fig. 7g, h) and previous reports indicating increased neurogenesis after blocking PFK function^102^ supports this hypothesis. Further investigations on these two and other candidate genes could link the neurogenic outcome to the amelioration of the AD pathology in mammalian brains based on our mouse and zebrafish experiments and human AD cohort results and can provide metabolic intervention strategies for enhancing neurogenic outcome and reducing reactive gliosis in human brains.

It is also noteworthy to mention that previous studies have conflicting findings regarding the role of Ngfr in AD pathology. Although the beneficial role of Ngfr/p75Ntr signaling is shown^61,103–105^, studies proposing a negative role of p75Ntr signaling on AD pathology also exist^106,107^. These studies widely differ in the animal models, in the promoters to express the receptor or its different variants and in the analytical methods with their statistical approaches. Therefore, the cell-specific roles of Ngfr and its relationship to long-term AD pathology could be context-dependent. The astroglial function of Ngfr could be different that the neuronal one and the spatiotemporal regulation exerted by Ngfr could make vast differences in the AD pathology. In our study, we relate the induced Ngfr activity to pro-neurogenic and anti-reactive state of astroglia, which is a cell type that have a multitude of functions in their vastly heterogenous cell states throughout the lifespan of humans^93^. Recently, we showed that astroglial end-feet need to be retracted from the blood vessels to enhance efficient clearance of amyloid aggregates, and genetic mutations in humans alter this ability^28^. While astroglia undergo proliferation, they retract their end-feet^94,108–111^ and this could help enhance toxic protein clearance, potentially linking pro-neurogenic potential of astroglia to reduced AD pathology. Longitudinal studies in animal AD models and in human AD cohorts could help scrutinize the versatile cellular aspects of astroglia in modifying AD pathology in the future. Finally, our study identified *Slc22a17* as a potential drug target to design therapies for interventions that will aim to enhance neurogenesis in AD.

Our study has strengths and limitations. Our findings are strong in terms of providing a previously unidentified mechanistic link for the astrocyte to toggle between reactive state and pro-neurogenic state, which favors neurogenesis and concomitant reduced AD pathology burden. Additionally, we provide an evolutionarily conserved mechanism for NGFR signaling (from zebrafish to mammals) in defining a pro-neurogenic astroglia. We provide evidence for the antagonistic effects of NGFR signaling on LCN2 and reactive gliosis using two mammalian species – human and mouse and identified that LCN2 expression levels correlate with increased amyloid burden in human brains. We utilized transcriptomics, spatial proteomics, in vivo functional knockdown studies, cell labeling and tracing as sensitive tools and identified a potentially druggable receptor, Slc22a17. We functionally showed that Ngfr weighed co-expressing gene *PFKP* mimics the beneficial effects of Ngfr on neurogenesis. We also provided evidence that Ngfr signaling could be a determinant of neurogenic outcome in humans, which is reduced with age. Finally, we provide candidate intracellular signaling mechanisms partaking in how the balance between reactive and neurogenic astrocyte physiology could be regulated. We also note the limitations of our study. We performed transient knockdowns in vivo, and transgenic animal models can be used in the future for measuring the sustained effects. Additionally, Tau pathology mouse models could be used to determine the long-term effects of neurogenesis and NGFR signaling on Tauopathies. We used a ubiquitous promoter for the viral gene expression because we sought for tracing the transduced cells for long-term. Therefore, astroglia-specific promoters would not be feasible. Our initial transduction mainly labels astrocytes and neurogenic studies as well as knockdown analyses were performed specifically on astroglia, but we cannot exclude that the nearby cells could also contribute to the overall pathological alterations in the long-term. Based on our observations, the transduction rate of non-astroglial cells is rather low and would not affect our conclusions drawn, yet in the future, cre-lox based cell specific recombination strategies could be employed for cell-specific loss or gain-of-function. Amyloid and Tau pathologies are related to cognitive decline^112,113^, yet the molecular mechanisms of how amyloid pathology directly or in turn by affecting the Tau pathology alters the cognitive capacity is an ongoing research focus^75^. In our model, we focused on how astroglial reactivity could be modulated to favor for more neurogenesis and the molecular mechanisms that might underlie this response, which might be related to the cognitive outcomes. However, the long-term behavioral assessment of the Ngfr-transduced AD mouse model would be an important measurement to test potential relationships of the neurogenic outcome to cognitive changes. We note that reduction in pathological burden in AD may not necessarily mean cognitive betterment, yet several studies demonstrated that enhanced neurogenesis could induce better cognitive task management and reduced cognitive decline^44,114–116^, and therapeutic intervention to amyloid accumulation has positive cognitive outcomes by reducing the cognitive decline in patients^117^. The association of Ngfr signaling to long-term behavioral readouts will be informative and important for addressing these questions.

## Methods

### Inclusion and ethics statement

All animal experiments were performed in accordance with the applicable European regulations and approved by the responsible authority (Landesdirektion Sachsen Germany and TU Dresden-Kommission für Tierversuche) under license number TVV87/2016. Animals were handled with extreme caution to reduce suffering and overall animal numbers. Human brain samples were obtained from New York Brain Bank within institutional regulations of Columbia University. The analyses conducted at Mayo Clinic were approved by the appropriate Mayo Clinic Institutional Review Board. Primary human astrocytes were purchased from a commercial source, and their usage was approved by the official regulatory boards. The use of primary human astrocytes was exempt from human subject regulations as the donors were deidentified, and the researchers in this study were not involved in obtaining the cells from the donor.

### Animal maintenance

Mice were kept under pathogen-free conditions in strict 12 h alternating light and dark cycle, with standard mouse food (chow) and water *ad libitum*. They were group-housed in standard ventilated cages prior to the experiments, and were kept in individual cages after surgical procedures. Fixed gender mice, aged between 52–56 weeks were used for the experiments unless stated otherwise. B6.Cg-Tg(APP695)3DboTG(PSEN1dE) mice were obtained from Jackson Laboratories (Bar Harbor, ME, USA) and were maintained as a heterozygous breeding colony.

### Lentiviral construct and production

In this study, we have designed and used p6NST90-hUb-mNgfr-T2A-mCherry-Lv16 (Lv16) and p6NST90-hUb-mCherry-Lv13 (Lv13) for lentiviral production (Fig. Supplementary 1). These lentiviral plasmids were constructed using a second-generation HIV based lentiviral system, comprising of 3 plasmids, (a) p6NST90 - transfer vector plasmid that contains the gene of interest (mNgfr and mCherry)^118,119^; (b) pCD/NL-BH - packaging plasmid that contains the Gag, Pol, Rev and Tat genes; and (c) pczVSV-Gwt - envelope plasmid that encodes the VSV-G protein. Mouse *Ngfr* under the mammalian promoter from the human ubiquitin C gene and mCherry, separated by T2A, were cloned in the backbone of the HIV transfer vector to generate the Lv16 construct. Similarly, Lv13 was constructed without mouse *Ngfr* as a control vector (see Supplementary Table 1 for primer details).

To produce viral particles, we co-transfected the packaging cells (HEK293T cells, ATCC Catalog number CRL-3216) with the vectors pczVSV-Gwt and pCD/NL-BH and the transfer vector p6NST90, in a 10 cm dish with 8 ml of DMEM (10% heat-inactivated FBS, 1% Pen/Strep), 18–21 dishes per condition. 5 μg of each plasmid was mixed in 1 ml prewarmed blank DMEM (without FBS and pen/strep) and combined with 1 ml of blank DMEM containing 45 μl of polyethylenimine (PEI) before adding to the HEK293T cells. At 48 h post-transfection, the medium from the transduced cells was collected, filtered, and concentrated by several rounds of ultracentrifugation. The presence of mCherry expression was verified by fluorescent microscopy.

### Primary human astrocytes and mouse neural stem/progenitor cells culture

Using complete astrocyte medium (ScienCell Research Laboratories - SRL, Catalog Number 1801), supplemented with 5% fetal bovine serum (SRL, Catalog Number 0010), 1% astrocyte growth supplement (SRL, Catalog Number 1852), and 1% penicillin/streptomycin solution (SRL, Catalog Number 0503), primary human astrocytes (pHA, SRL, Catalog Number 1800) at passage number 1 were sub-cultured to obtain passage 2 pHAs. These stocks were used for all relevant experiments in this study. Mouse neural stem/progenitor cells (NSPCs) were isolated from the dentate gyri of 3-month-old WT mice as previously described^33,120,121^. Briefly, DGs were dissected out of the hemispheres of the freshly isolated adult mice brains on ice in PBS containing Pen/Strep. Using a scalpel, tissues were minced followed by enzymatic digestion with the Neural Tissue Dissociation kit from Miltenyi Biotec as per the manufacturer’s instructions. Following the dissociation, the cell suspensions were cultured in PDL/Laminin coated 25 cm^2^ flask using Neurobasal medium (GIBCO, Life Technologies), supplemented with 2% B27 (Invitrogen), 1x GlutaMAX (Life Technologies), 50 units/ml penicillin/streptomycin, 20 ng/ml EGF (Peprotech, AF-100-15), and 20 ng/ml FGF (Peprotech, AF-100-15). Cells were kept in an incubator with a 5% CO2/95% air atmosphere at 37 °C, and media was exchanged every 48 h. Only passages 7-10 were used during the experiments. Primary human astrocytes^122^, were treated with 1.5 mM of PFK allosteric regulator sodium citrate^59,123^ or PBS for a week, provided together with complete astrocyte medium in a 24-well format. Followed by PFA fixation and immunostaining.

### Biohybrid-hydrogels 3D culture and Lipocalin-2 treatment

Primary human astrocytes (SRL, Catalog number 1800) were encapsulated in biohybrid-hydrogels as previously described^122^. 1.Transfer the frozen pHAs vial from the cryogenic storage to a 37 °C water bath immediately. Briefly, the cell concentration was adjusted to 5000 cells/cm2 of culture-ware, and pHAs were incubated for 1 day in a cell culture incubator at 37 °C with 5% CO_2_. 10 µl starPEG-Heparin hydrogels were created with a concentration on 4 million cells per ml and were treated with 200 ng/ml Lipocalin-2 or PBS in a complete astrocyte medium for one week. After two weeks of culture, hydrogels were fixed using 4% PFA and were stained for relevant markers.

### Generation of Lv13 and Lv16-transduced human astrocytes

Lv16-p6NST90-hUb-mNgfr-T2A-mCherry and Lv13-p6NST90-hUb-mCherry human astrocytes cell lines were generated using pHA (SRL, catalog number 1800) of passage 3, which were transduced with respective viral vectors. For transduction, pHAs were seeded in a 24-well plate and were cultured until cells reached 70-80% confluency using a complete astrocyte medium. The respective viruses were then added to each well and cells are incubated for 24 h at 37 °C in an incubator with a 5% CO2/95% air atmosphere. After 24 h, the media is exchanged, and cells are allowed to grow until they were 90% confluence. Cells supernatant was checked for viral load after 72 h of infection and virus particle-free cells were passaged to a bigger vessel. Cells from overall passage 6 were used for all experiments.

Slc22a17 and control morpholino on Lv13 and Lv16 mouse neural stem/progenitor cell lines: mouse NSPCs from passage 7 were used to generate the Lv16-p6NST90-hUb-mNgfr-T2A-mCherry and Lv13-p6NST90-hUb-mCherry mouse NSPCs cell line. Mouse NSPCs were cultured in a PDL/Laminin coated 25 cm^2^ flask at 10,000 cells per cm^2^ concentration using Neurobasal plus medium (GIBCO, Life Technologies), supplemented with 2% B27 (Invitrogen), 1x GlutaMAX (Life Technologies), 50 units/ml penicillin/streptomycin, 20 ng/ml EGF (Peprotech, AF-100-15), and 20 ng/ml FGF (Peprotech, AF-100-15). Lv16 and Lv13 virus were then added to each flask and cells are incubated for 24 h at 37 °C in an incubator with a 5% CO2/95% air atmosphere. Media was changed after 24 h and cells are allowed to recover and grow until they were 70–80% confluence (approx. 4 days, media change at every 48 h). 5 μM of control and *Slc22a17* morpholino oligos were added to respective cell line flasks and were incubated for 24 h. Cells were washed after 24 h with fresh media.

### Stereotaxic injections of lentiviral vectors, Lipocalin-2, and neural stem cells

The stereotaxic injection procedure was carried out as previously established protocol^124^. Briefly, during the entire surgery, the mice were anesthetized using a mix of oxygen and isoflurane (49:1) (Baxter – HDG9623) flow and placed on a pre-warmed heat-pad to prevent hypothermia. The head was immobilized with the help of ear bars and the eyes were with a protective ointment to avoid cornea dehydration. To minimize any possible pain after the surgery, an analgesic was subcutaneously injected prior to the procedure. The hippocampal injection coordinates were ±1.6 mm mediolateral, −1.9 mm anterior-posterior, and −1.9 mm dorsoventral from the Bregma, where the virus was dispensed at 200 nl/min speed. After the injection, the capillary was slowly retracted, followed by the release of the ear bars and stitching of the injection site.

For the viral injections of p6NST90-hUb-mNgfr-T2A-mCherry-Lv16 (Lv16) or p6NST90-hUb-mCherry-Lv13 (Lv13), 1 µl of each virus was injected into either hemisphere. For the Lcn2 injection experiment, 1 µl of 10 ng/µl Lcn2 solution was injected into the right hemisphere and 1 µl vehicle solution into the left. For the injection of morpholino-treated cells, after 24 hours of morpholino treatment cells were lifted using Accutase (Gibco, A11105‐01), immediately followed by a cranial injection to avoid keeping them on ice for longer than 1 h. Each hemisphere of WT mice was delivered with 1×10^5^cells/µl of cell suspension at a speed of 200 nl/min using the Nano-injector system. At 3 days post-injection, mice were sacrificed either via cervical dislocation followed by hippocampi extraction or via transcardial perfusion to isolate the whole brain for further processing.

### BrdU labeling and tissue preparation

To label proliferating cells, mice were injected intraperitoneally (IP) with BrdU (50 mg/kg) 3 times on the day of cranial injection, 9 h apart, followed by an IP every 24 h until the end of experiment. Mice were sacrificed by an overdose of Ketamine/Xylazin (0.25 mL per 25 g of body weight) mixture and perfused transcardially with NaCl (0.9% w/v) followed by 4% paraformaldehyde (PFA). Brains were harvested and post-fixed in 4% PFA at 4 °C overnight. For cryopreservation of the fixed tissue, brains were transferred to a 30% sucrose solution for 2–3 days. Coronal sections with a thickness of 40 mm were cut using a sliding microtome (Leica SM2010) cooled with dry ice. Free floating sections were collected in 6 consecutive series and stored in cryoprotection solution (CPS; 25% ethylene glycol, 25% glycerol in 0.1 M phosphate buffer pH 7.4) at −20 °C. Every sixth section of each brain was pooled in one series for immunohistochemistry.

### Human brain tissue samples

Autopsy brain samples were obtained from the New York Brain Bank at Columbia University Medical Center. The demographics and postmortem neuropathological findings of human cases identified in the Columbia University Alzheimer’s Disease Research Center Neuropathology Core and used in this study are listed in Supplementary Table 2. These specimens were obtained by consent at autopsy and have been de-identified and are IRB-exempt to protect the identity of each patient. Formalin-fixed paraffin-embedded (FFPE) specimens were sectioned by the Histology Service at Columbia University Medical Center. Immunohistochemistry was performed on 6 μm paraffin-embedded sections as previously described^28,125,126^. Briefly, sections were passed through Xylene (Millipore Sigma, 1.94600) and decreasing methanol (Millipore Sigma, 1424109) stages to distilled water for deparaffinization; treated with citrate buffer (pH: 6,0), permeabilized with 0.2% PBST (T:tween20, Roche, 11332465001); blocked with 10% normal goat serum (Millipore Sigma, NS02L); treated with primary antibody mixes at 4 °C overnight, secondary antibody for 30 min at room temperature; and nuclear counterstained with DAPI (Millipore Sigma, D9542).

### Fluorescence immunohistochemistry

Mouse tissue: Prior to immunohistochemistry, the free-floating sections were washed in PBS three times, blocked in 10% Donkey or Goat Serum, 0.3% Tween 20, 1x PBS solution for one hour at room temperature. In the case of Dcx and BrdU staining, antigen retrieval was performed in 2 N HCl for 30 min at 37 °C followed by 3x washing in PBS. Primary antibodies (4G8, Aldoc, AT8, Bdnf, B-III-Tubulin, BrdU, Dcx, Gfap, Hopx, Iba1, Lcn2, mCherry, Map2, Mcm7, NeuN, Ngf, Ngfr, Olig2, proBdnf, pTauS199, S100β and Slc22A17) were diluted in PBS together with 3% Goat/donkey serum and 0.3% Tween 20 and incubated overnight at 4 °C with the sections. This was followed by washing with PBS 3 times within an hour and incubating for 4 h at room temperature with the correct secondary antibody conjugated with a desired fluorophore. After short wash samples are then incubated in 4,6-diamidino-2-phenylindole (DAPI) diluted in PBS for 15 min. Additional steps of washing were done, and samples are mounted on the charged glass slides. After mounting slides are left to dry and covered with a coverslip using Aqua-Poly/Mount (Polysciences Europe GmbH). Biohybrid-hydrogels: fixed hydrogels were blocked and permeabilized using 10% Goat Serum, 0.3% Tween 20, and 1x PBS solution for 1 h at room temperature, followed by primary and secondary antibody treatments (in 3% Goat serum 0.3% Tween 20, and 1x PBS). 2D-cell cultures: mouse hippocampal astrocytes were fixed with 4% PFA (15 min) followed by blocking and permeabilization, using 10% Donkey Serum, 0.3% Tween 20, and 1x PBS solution for an hour at room temperature, followed by overnight primary antibody treatment and 2 h secondary antibody treatments (in 3% donkey serum 0.3% Tween 20, and 1x PBS). See Supplementary Table 1 for antibody information, Supplementary Table 2 for information on the human brain samples.

### Imaging and quantifications

Spinning Disc Zeiss Axio Observer.Z1 microscope (Oberkochen, Germany) equipped with ZEN software (version blue edition, v3.2, company, Carl Zeiss, Jena, Germany) was used to acquire the fluorescence images of the 40-µm thick mouse brain tissue sections (along with the complete ventral dorsal extent of the DG at 20x magnification) and the biohybrid hydrogels (5 images per hydrogel at 10x magnification, image acquisition dimensions were 704, 999, 300 µm, respectively, with a step size of 2 µm). Human brain sections were imaged with Zeiss LSM 800 scanning confocal microscopes with Airyscan super-resolution module (Oberkochen, Germany). 2D cell cultures were imaged using Operetta CLS microscope, at 20x magnification. Images were analyzed using ZEN (v3.2, Carl Zeiss, Jena, Germany) or ImageJ (v1.53, NIH, Bethesda, MD, USA, https://imagej.nih.gov/ij/) or Arivis Vision 4D (v4) software. BrdU/GFAP, BrdU/DCX/mCherry, and BrdU/GFAP/mCherry cells were manually counted in every sixth section (240 mm apart) and were normalized to the area (measured via Arivis vision 4D software) of the granular layer of the dentate gyrus present through the ventral dorsal axis of each hemisphere (Figs. 2c, f, i, l–n, 3d, g, 4k, Supplementary Fig. 5, Supplementary Data 4). In the case of morpholino-treated cells injection, BrdU/DCX/mCherry triple-positive cells were manually counted, only the sections with at least one mCherry positive cell in the granular layer were considered for analysis (Fig. 4n). Quantification of amyloid plaques (4G8 staining) and phosphorylated Tau-S199 was done via intensity measurement (using Zen v3.2 software), the total intensity was normalized to the area dentate gyrus with proper background noise correction (Fig. 5i, j and Supplementary Data 9). 4G8 surface area quantification (Fig. 5k), AT8 staining area quantification (Fig. 5m) was performed using Fiji/ImageJ. Volume analysis of the hydrogel encapsulated GFAP cells (Fig. 6g) and LCN2 expression in human brain sample (Fig. 6e), quantification of mCherry, GFAP, MAP2, Slc22A17, Beta-III-tubulin positive cells in hydrogels (Figs. 6i, 7h and Supplementary Figs. 1, 6), were performed in an automated manner using the Arivis vision 4D pipeline (pipelines available upon reasonable request).

### Single cell sequencing and analyses

Mouse hippocampi were isolated as described^33^ and cells were kept on ice until dissociated into cells which were used for the single-cell sequencing. Single cell sequencing was performed as described^19,20,33^. Sequencing dataset is available on NCBI GEO (https://www.ncbi.nlm.nih.gov/geo/) with accession number GSE189626. We performed all analyses with R.4.0 and Seurat V4. A Seurat object was generated for each dataset, the data were normalized with NormalizeData and 500–2000 variable genes were identified. Data were scaled and nCount_RNA (nUMI) were regressed out. The first 20 PCA were determined, clusters were identified using resolution of 1 and UMAP was calculated for 2D visualization. Data scaling, cluster identification and UMAP detection was performed as described before^20^. To identify main cell types, we used known marker genes and top marker gene of each cluster. The marker genes were identified using the Seurat function “FindAllMarkers” with only.pos = T. Then, top 20 markers of each cluster were identified and heatmaps were generated. Human organoid, fetal brain and adult brain single cell sequencing clustering, heat map generation and tSNE plots were generated as described^19,31^.

### NanoString GeoMx digital spatial profiling (DSP)

Using DSP, we performed a multiplexed and spatially resolved profiling analysis on Lv16-p6NST90-hUb-mNgfr-T2A-mCherry and Lv13-p6NST90-hUb-mCherry injected contralateral hemispheres of WT and APP mice. DSP technology uses antibodies with UV photocleavable indexing oligos for protein profiling within the selected regions of interest (ROIs). Using the slide preparation protocol from NanoString Technologies, Inc (Seattle, WA, USA), 5-µm-thick FFPE sections of WT and APP mice brains were prepared from the viral injected site. Morphology markers for visualizing the neural cells (GFAP, AF594), immune cells (Iba1, AF647), mCherry (AF532), and nucleic acids (AF488), were applied for 1 h at room temperature prior to being loaded on the GeoMx Digital Spatial Profiler (NanoString Technologies, Inc). As shown in Fig. 5a, based on fluorescence imaging, ROIs (200–600 µm in diameter) within the DG areas were chosen for multiplex profiling. The DSP exposed each ROI to a 385-nm light to release the indexing oligos, and the photocleaved oligos were transferred into a microwell. The DSP sequencing data were processed using the GeoMx DSP analysis suite (GEOMX-B0007). Reads were normalized to signal-to-noise ratio (Fig. 5b and Supplementary Data 7, 8).

### Human Alzheimer’s disease versus control differential expression analysis

We compared the conditional-quantile-normalized^127^ gene expression levels between AD and control brains in the AMP-AD datasets^49,51–53^ using a multiple linear regression model adjusting for the biological and technical variables (age, sex, sequencing flow-cell, RIN, tissue source, *APOE4* allele dosage). Multiple testing was adjusted using false discovery rate (FDR). The cell-intrinsic DEG (CI-DEG) analysis^54^ and the weighted gene co-expression network analysis (WGCNA)^55,56^ were performed.

### Statistical analyses

Data analysis was performed using Prism software (Version 8, GraphPad Software, Inc). Results were expressed as mean ± standard error of the means (SEM). Statistical significance was determined using *t* test when the experiment contained two groups (parametric test when normal distribution was predicted and nonparametric test with cumulative distribution comparison by using Kolmogorov-Smirnov test), and ANOVA when comparing more than two groups (ordinary ANOVA when normal distribution was predicted, and non-parametric test with Kruskal-Wallis test in case of non-Gaussian distribution). Bonferroni, Sidak, Tukey, Dunnett, original Benjamini and Hochberg, or Benjamini, Krieger, Yekutieli FDR methods were used for multiple comparison. Information provided in the text wherever applicable. The level of statistical significance was displayed as *p < 0.0332, **p < 0.0021, ***p < 0.0002, and ****p < 0.0001.

### Reporting summary

Further information on research design is available in the Nature Research Reporting Summary linked to this article.

## Supplementary information

Supplementary Table 1

Supplementary Table 2

Supplementary Information

Reporting Summary

Supplementary Data 1

Supplementary Data 2

Supplementary Data 3

Supplementary Data 4

Supplementary Data 5

Supplementary Data 6

Supplementary Data 7

Supplementary Data 8

Supplementary Data 9

Supplementary Data 10

## Data Availability

All data and relevant accession numbers are available in the main text or the supplementary materials. Raw data for mouse single cell sequencing is available on GEO (accession number GSE189626). Supplementary Materials: Supplementary Figs. 1 to 12, Supplementary Tables 1 to 2, Supplementary Data 1 to 10. AMP-AD data can be accessed via the AD Knowledge Portal. For the AMP-AD accession numbers, please use the following IDs: bulk DEGs: ROSMAP: syn3219045; Mayo: syn5550404; MSBB: syn3159438. CI-DEG: syn22228843; WGCNA: syn5550404. The AD Knowledge Portal is a platform for accessing data, analyses and tools generated by the Accelerating Medicines Partnership (AMP-AD) Target Discovery Program and other National Institute on Aging (NIA)-supported programs to enable open- science practices and accelerate translational learning. The data, analyses and tools are shared early in the research cycle without a publication embargo on secondary use. Data is available for general research use according to the following requirements for data access and data attribution (https://adknowledgeportal.synapse.org/DataAccess/Instructions).

## Code availability

Images were analyzed using ZEN (v3.2, Carl Zeiss, Jena, Germany), ImageJ (v1.53, NIH, Bethesda, MD, USA, https://imagej.nih.gov/ij/) and Arivis Vision 4D (v4) software. Single cell data was analyzed with R package R.4.0 and Seurat v4. GraphPad Prism version 8 was used for statistical analyses. All codes are available upon reasonable request.
