# Supplementary material for: Nerve growth factor receptor (Ngfr) induces neurogenic plasticity by suppressing reactive astroglial Lcn2/Slc22a17 signaling in Alzheimer’s disease
Source: NPJ Regen Med. 2023 Jul 10;8:33. doi: 10.1038/s41536-023-00311-5 (PMC10333226; doi:10.1038/s41536-023-00311-5)
Supplement: Supplementary file 3 — Supplementary Information [file 41536_2023_311_MOESM3_ESM.pdf]

Ngfr induces neurogenic plasticity by suppressing reactive astroglial Lcn2/Slc22a17 signaling in Alzheimer's disease

Tohid Siddiqui, Mehmet I. Cosacak, Stanislava Popova, Prabesh Bhattarai,
Elanur Yilmaz, Annie J. Lee, Yuhao Min, Xue Wang, Mariet Allen, Özkan İş, Zeynep T.
Atasavum, Natalia Rodriguez-Muela, Badri N. Vardarajan, Delaney Flaherty, Andrew F. Teich,
Ismael Santa-Maria, Uwe Freudenberg, Carsten Werner, Giuseppe Tosto,
Richard Mayeux, Nilüfer Ertekin-Taner, Caghan Kizil

Supplementary Information

Supplementary Figures 1-12 (provided in this PDF file)
Supplementary Tables 1-2 (provided online as .xlsx files)
Supplementary Data 1-10 (provided online as .xlsx files)

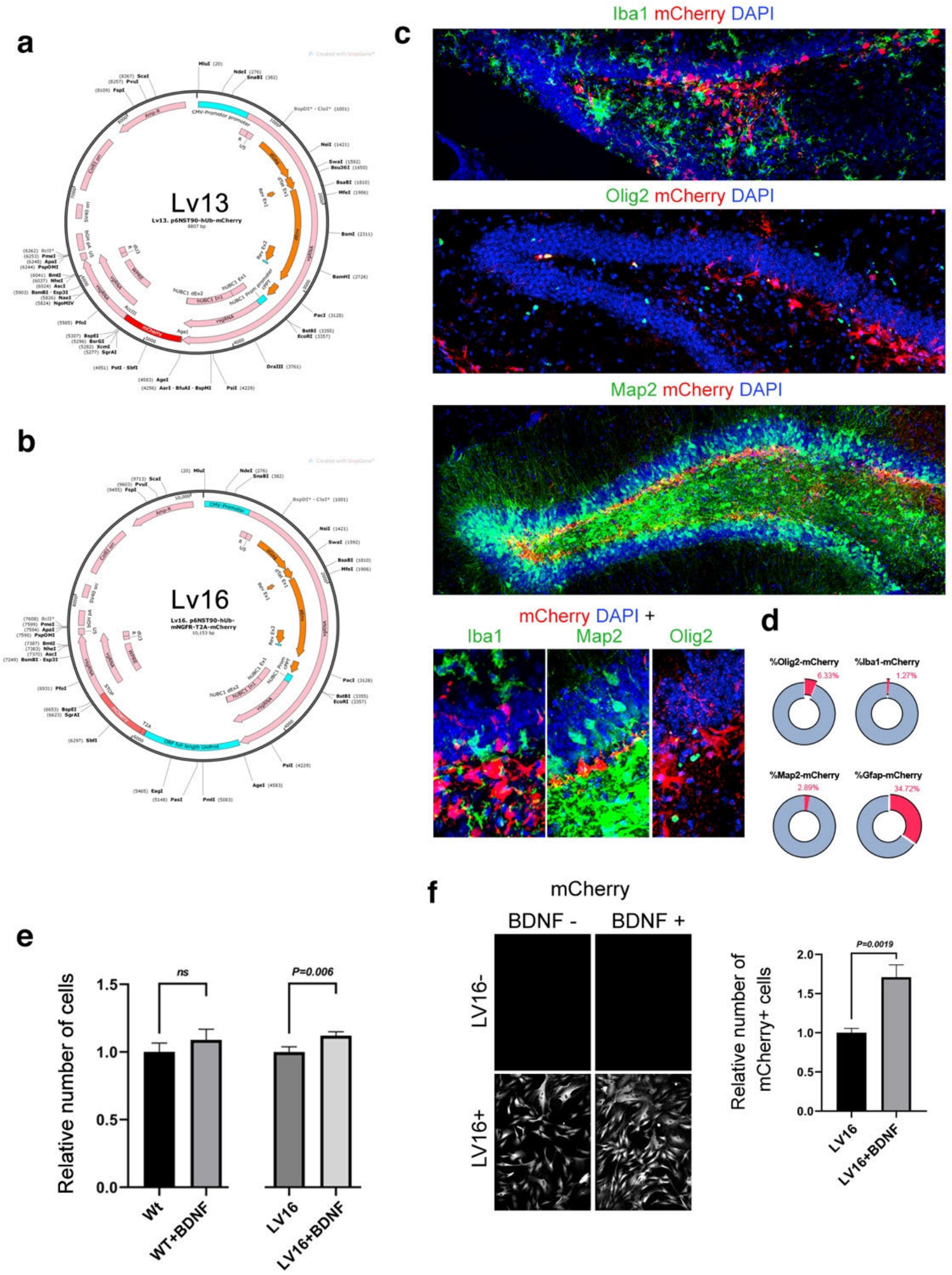

Supplementary Fig. 1. Maps of lentiviral vectors, in vitro functionality assay, single cell sequencing heat map. a. Lv13: CMV promoter driven mCherry. **b.** Lv16: CMV promoter-driven mouse Ngfr linked to mCherry with T2A self-cleaving peptide. **c.** Double immunostaining for Iba1 and mCherry for detecting transduced microglia, Olig2 and mCherry for detecting transduced oligodendrocytes, Map2 and mCherry for detecting transduced neurons. **d.** Quantification for percentage of the cell types transduced with Ngfr. **e.** Graphs show relative number of cells in culture dish after equi-number seeding of cells that are either untransduced (wt) or LV16-transduced. **f.** BDNF is added to the culture medium to activate Ngfr signalling. Only Lv16-transduced cells are responsive to BDNF. Staining image shows the mCherry -positive cells that increase in response to BDNF and Lv16. Graph indicates the number of mCherry-positive cells after equi-number seeding. Error bars represent standard error of the means.

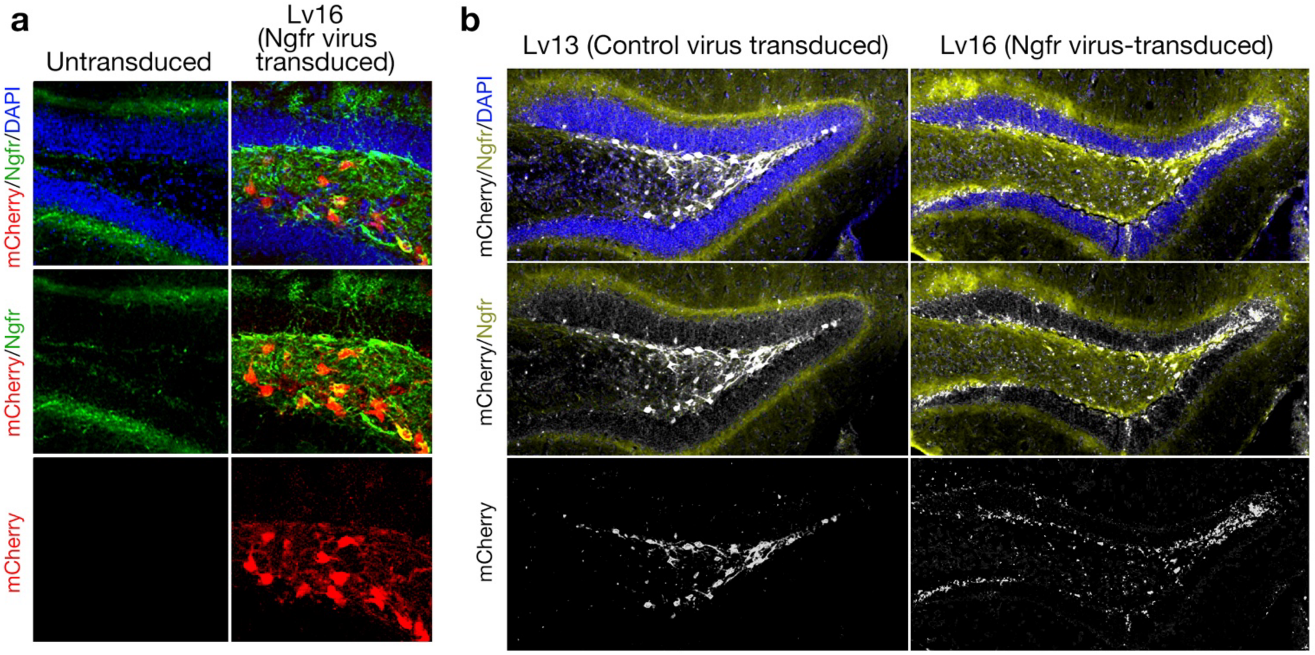

Supplementary Fig. 2. Single channel images for Ngfr transduction. a. Ngfr and mCherry immunostaining with DAPI counterstain in untransduced and Lv16-transduced mouse DG. **b.** Ngfr and mCherry immunostaining with DAPI counterstain in Lv13 and Lv16-transduced mouse DG.

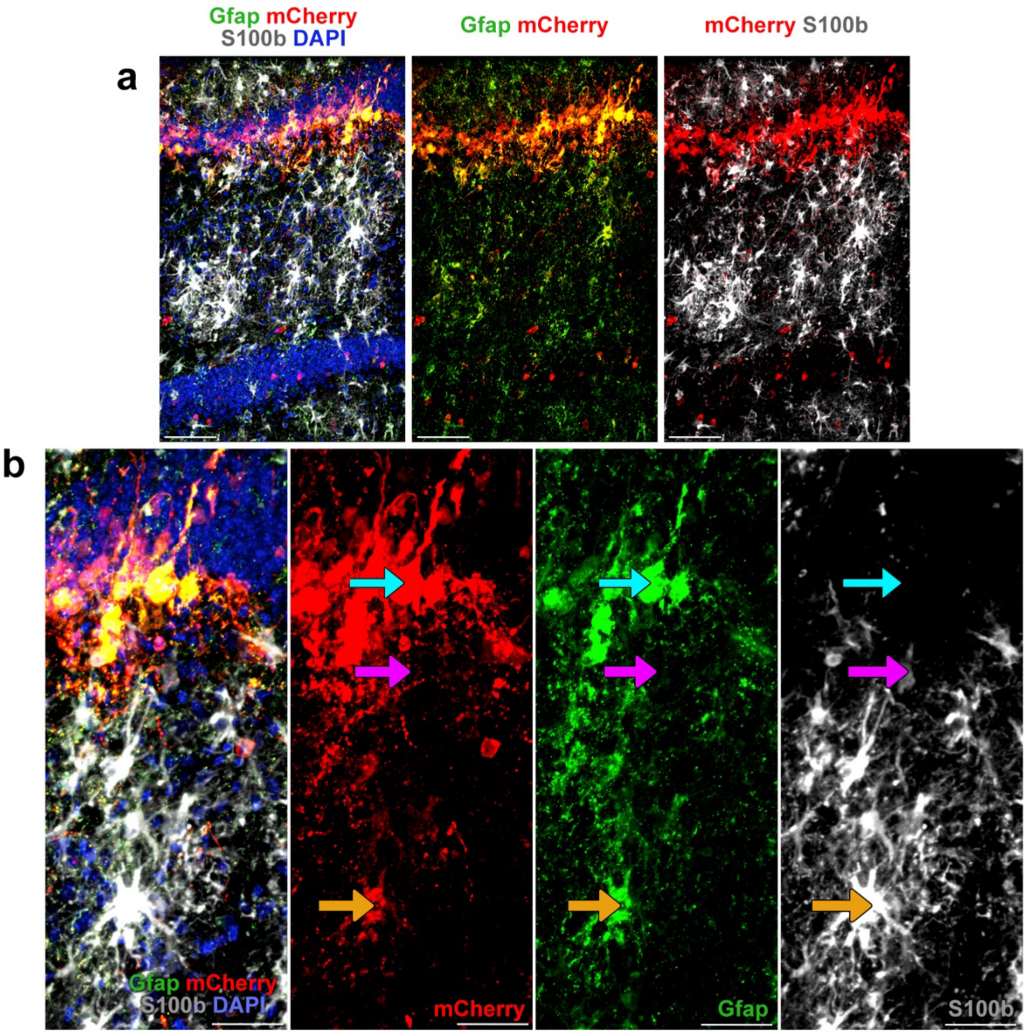

Supplementary Fig. 3. Ngfr transduction in Gfap+ astroglia. a. Gfap, mCherry and S100β immunostaining with DAPI counterstain in mouse DG. **b.** Higher magnification images. Cyan arrow: Gfap+/S100β-/mCherry+ astroglia, pink arrow: Gfap-/S100β+/mCherry- astroglia, yellow arrow: Gfap+/S100β+/mCherry+ astroglia. Scale bars equal 25 μm.

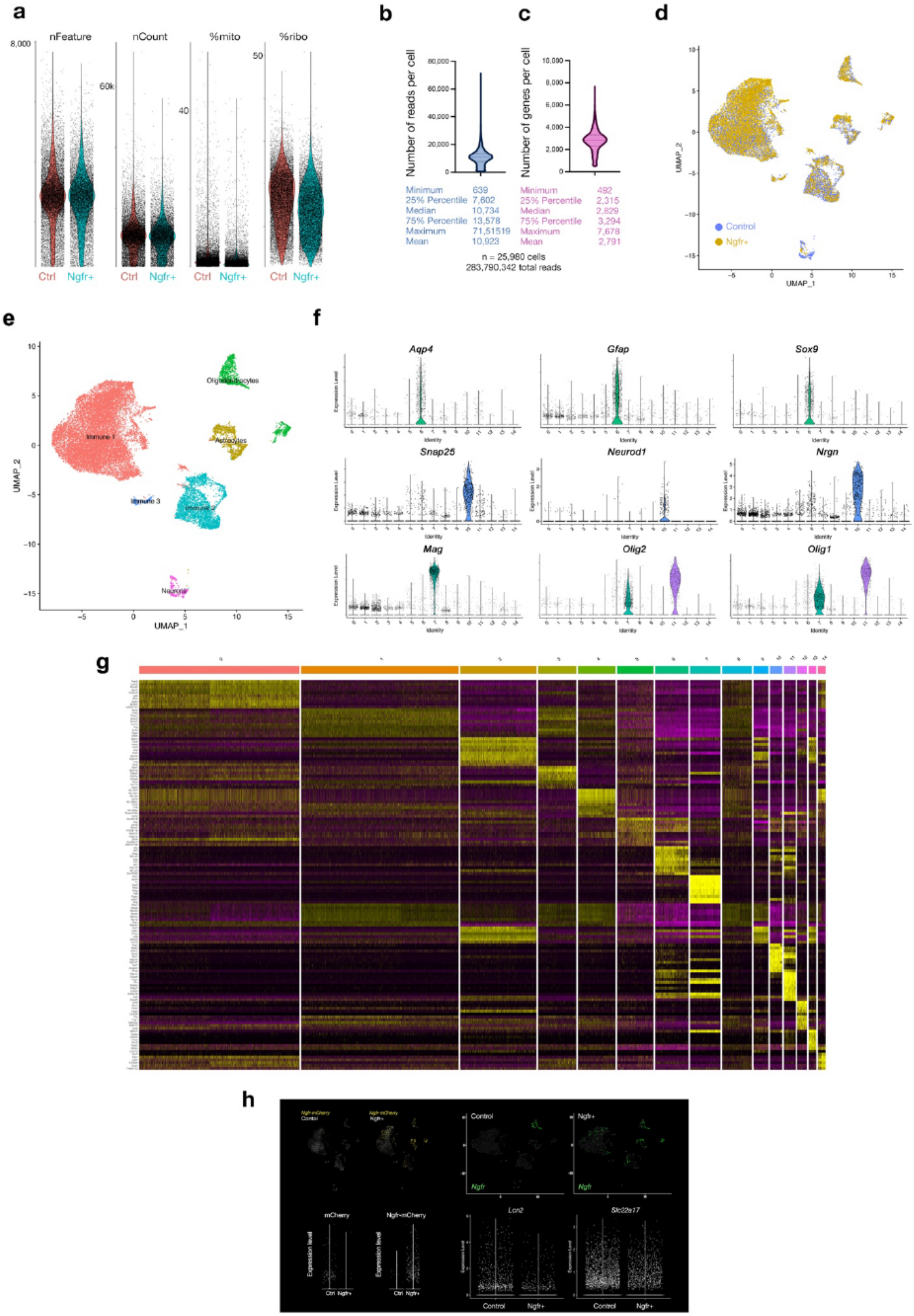
a
nFeature
nCount
%mito
%ribo
8,000
60k
40
50
Ctrl
Ngfr+
b
Number of reads per cell
Minimum 639
25% Percentile 7,602
Median 10,734
75% Percentile 13,578
Maximum 71,51519
Mean 10,923
c
Number of genes per cell
Minimum 492
25% Percentile 2,315
Median 2,829
75% Percentile 3,294
Maximum 7,678
Mean 2,791
n = 25,980 cells
283,790,342 total reads
d
UMAP_1
UMAP_2
Control
Ngfr+
e
Oligodendrocytes
Immune 1
Astrocytes
Immune 3
Immune 2
Neurons
f
Aqp4
Gfap
Sox9
Snap25
Neurod1
Nrgn
Mag
Olig2
Olig1
Expression Level
Identity
g
h
Control
Ngfr+
Ngfr
mCherry
Ngfr-mCherry
Expression level
Ctrl
Lcn2
Slc22a17

Supplementary Fig. 4. Single cell transcriptomics sample characteristics and quality control analyses. **a.** Distribution of number of genes sequenced per cell (nFeature), number of reads per cell (nCount), percentage of mitochondrial gene expression (%mito) and percentage of ribosomal gene expression (%ribo). **b.** Distribution of number of reads per cell. **c.** Distribution of number of genes sequenced per cell. **d.** tSNE plot for control and Lv16-transduced cells. **e.** tSNE plot for individual cell types. **f.** Violin plots for basic cell markers of astroglia, oligodendrocyte, and neurons. **g.** Heatmap for all cell clusters. **h.** Plots for *Ngfr-mCherry* expression in control and Ngfr-transduced hippocampus, *mCherry* and *Ngfr-mCherry* expression in control and Ngfr-transduced mouse DG, total *Ngfr* expression in control and Ngfr-transduced mouse DG, *Lcn2* expression in control and Ngfr-transduced mouse DG, and *Slc22a17* expression in control and Ngfr-transduced mouse DG. Error bars represent standard error of the means.

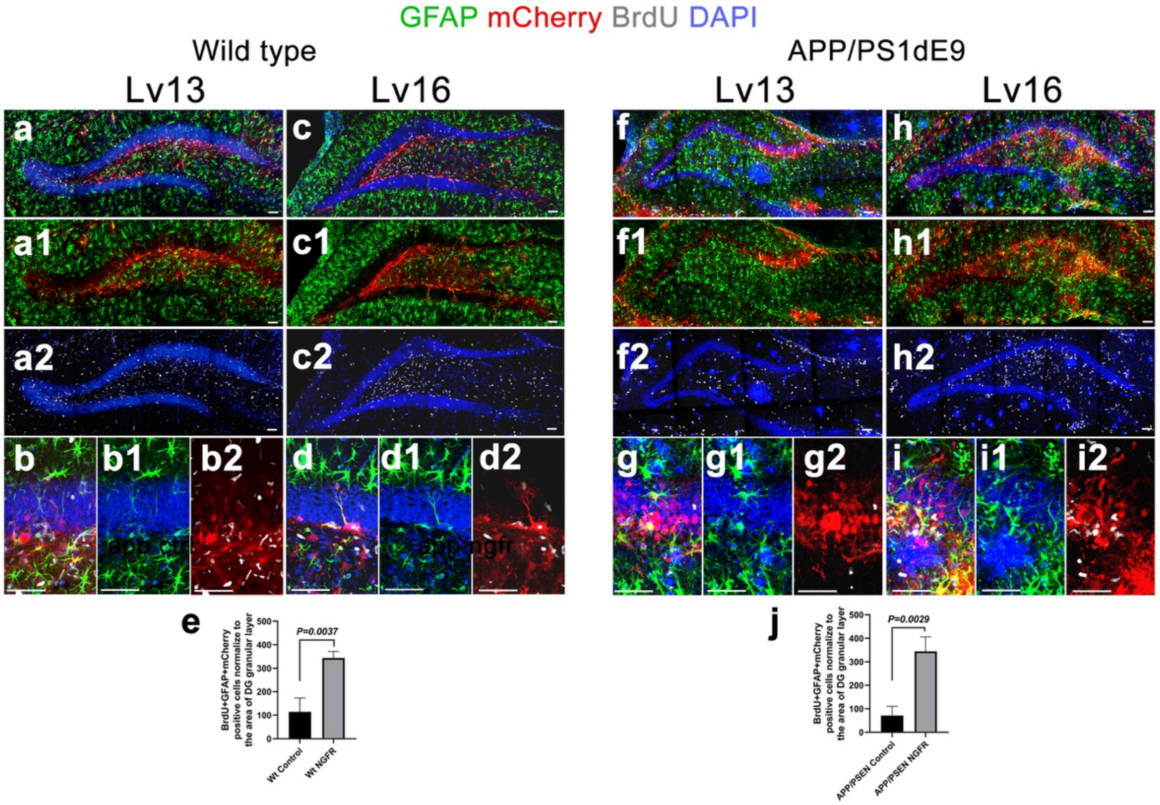

Supplementary Fig. 5. Lv16 transduction enhances astroglia proliferation in wild type and APP/PS1dE9 mouse model of Alzheimer's disease. a. GFAP, mCherry, BrdU triple immunostaining with DAPI counterstain in Lv13-transduced wild type mouse dentate gyrus. **a1.** DAPI and BrdU omitted from a. **a2.** GFAP and mCherry omitted from a. **b.** Higher magnification image of subgranular zone. **b1.** BrdU and mCherry omitted from b. **b2.** GFAP and DAPI omitted from b. **c.** GFAP, mCherry, BrdU triple immunostaining with DAPI counterstain in Lv16-transduced wild type mouse dentate gyrus. **c1.** DAPI and BrdU omitted from c. **c2.** GFAP and mCherry omitted from c. **d.** Higher magnification image of subgranular zone. **d1.** BrdU and mCherry omitted from d. **d2.** GFAP and DAPI omitted from d. **e.** Quantification graph for BrdU-mCherry-GFAP triple positive cells in Lv13 and Lv16-transduced brains. **f.** GFAP, mCherry, BrdU triple immunostaining with DAPI counterstain in Lv13-transduced APP/PS1dE9 mouse dentate gyrus. **f1.** DAPI and BrdU omitted from f. **f2.** GFAP and mCherry omitted from f**. g.** Higher magnification image of subgranular zone. **g1.** BrdU and mCherry omitted from g. **g2.** GFAP and DAPI omitted from g. **h.** GFAP, mCherry, BrdU triple immunostaining with DAPI counterstain in Lv16-transduced APP/PS1dE9 mouse dentate gyrus. **(h1)** DAPI and BrdU omitted from h. **h2.** GFAP and mCherry omitted from h. **i.** Higher magnification image of subgranular zone. **i1.** BrdU and mCherry omitted from i. **i2.** GFAP and DAPI omitted from i. **j** Quantification graph for BrdU-mCherry-GFAP triple positive cells in Lv13 and Lv16-transduced brains. Scale bars equal 100 μm. Error bars represent standard error of the means.

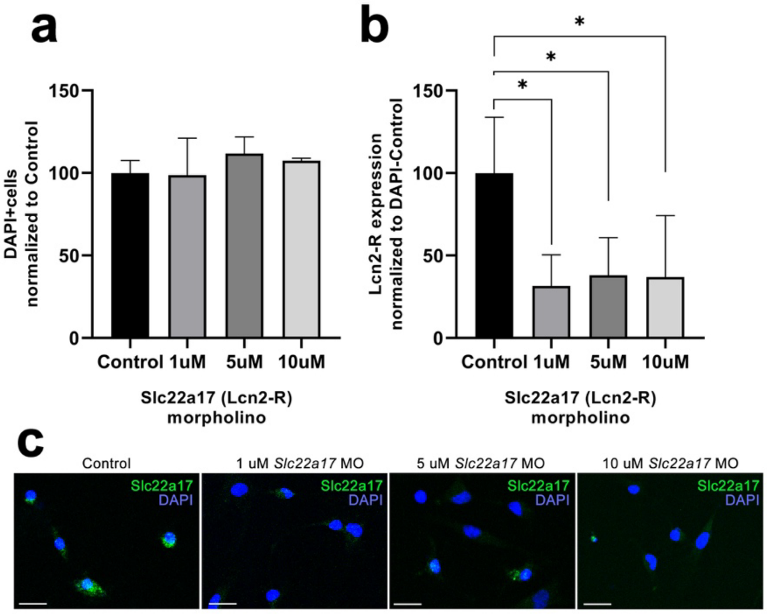

Supplementary Fig. 6. Effectiveness of *Slc22a17* morpholino. a. In vitro cell viability counts in astroglia that are untransfected or transfected with three doses of *Slc22a17* vivo morpholino (1-, 5- and 10 μM). **b.** Slc22a17 (Lcn2-R) protein expression (fluorescence units on immunolabeled cultures) normalized to DAPI in control and transfected astroglia. **c.** Slc22a17 immunostaining and DAPI counterstain in control (untransfected) astroglia and astroglia transfected with 1-, 5- and 10 μM vivo morpholino. Scale bars equal 25 μm. Error bars represent standard error of the means.

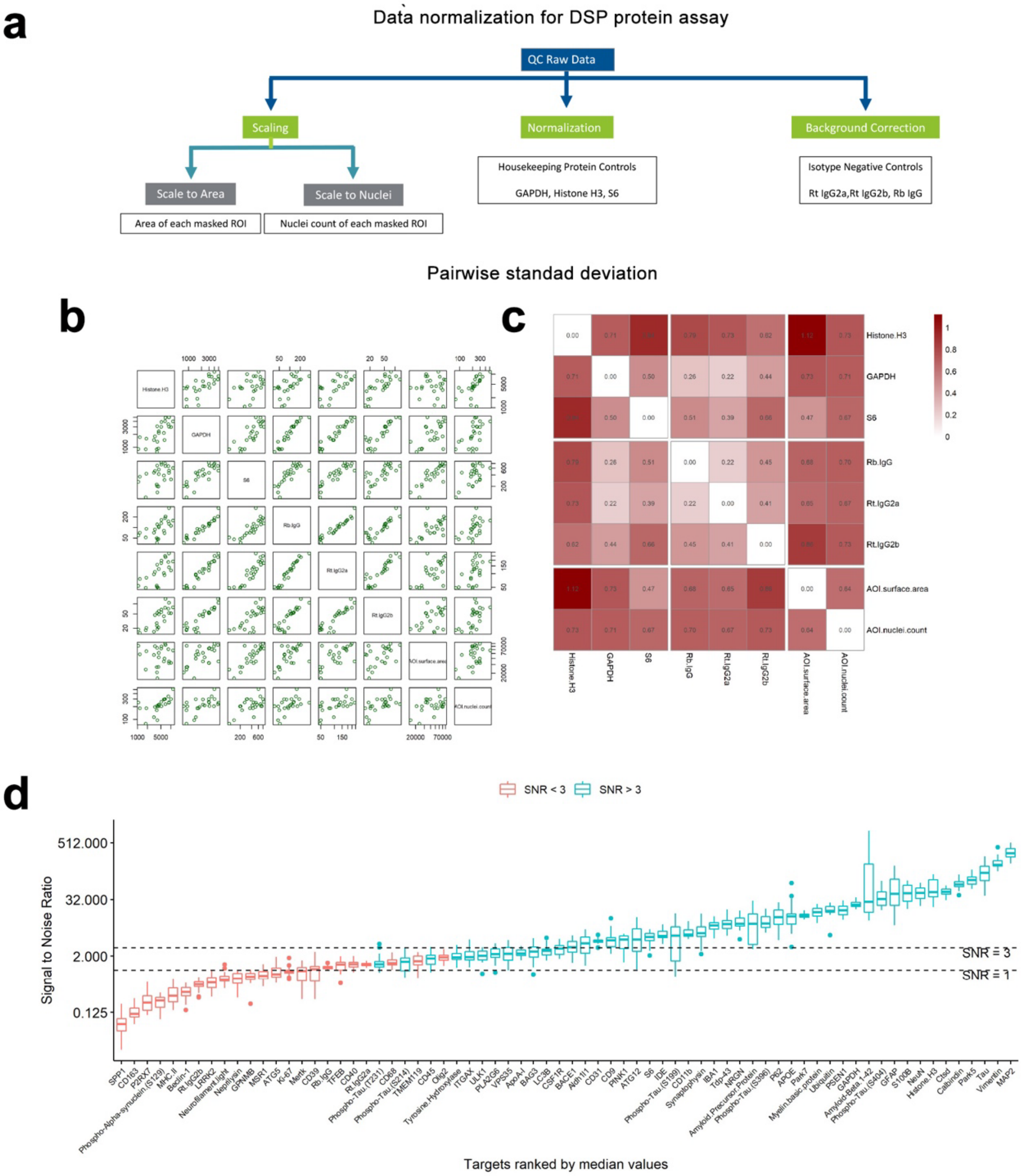

Supplementary Fig. 7. NanoString GeoMX quality control and normalization strategy. a. Data normalization options. **b, c.** Pairwise standard deviation analysis. b. Raw values plotted for spike-in normalization samples. c. Standard deviation heat map for surface area, nuclei count, housekeeping genes (Histone H3, GAPDH, S6) or signal-to-noise ratio (IgG, IgG2a, IgG2b). Signal-to-noise ratio normalization has the lowest standard deviation. **d.** Plot for targets ranked by median expression values normalized to signal-to-noise ratio. Error bars represent standard error of the means.

a

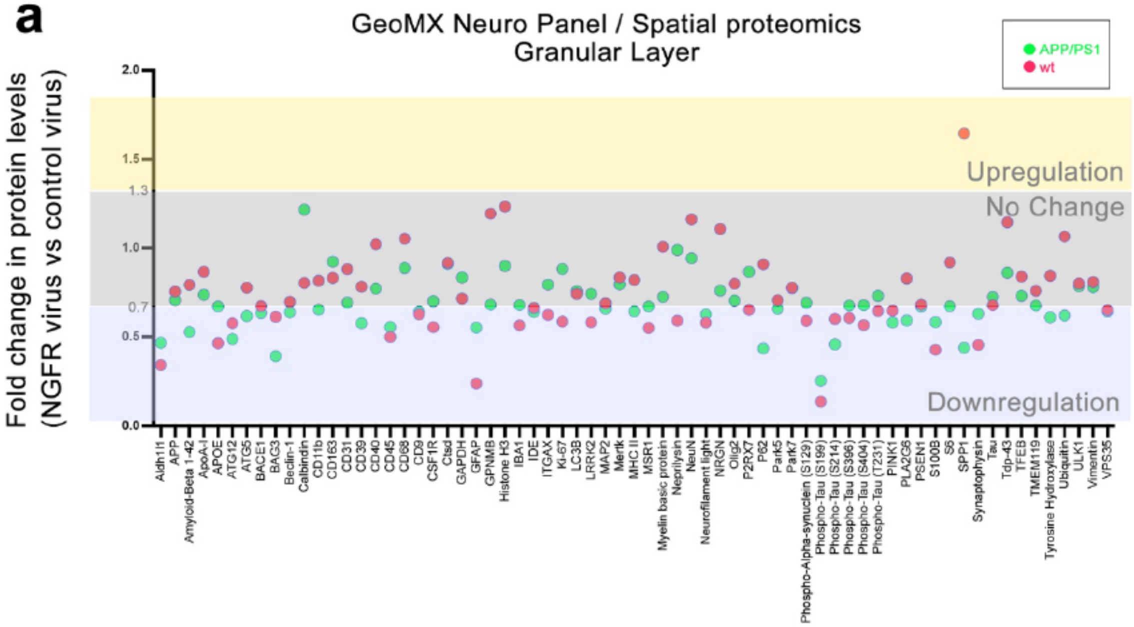

b

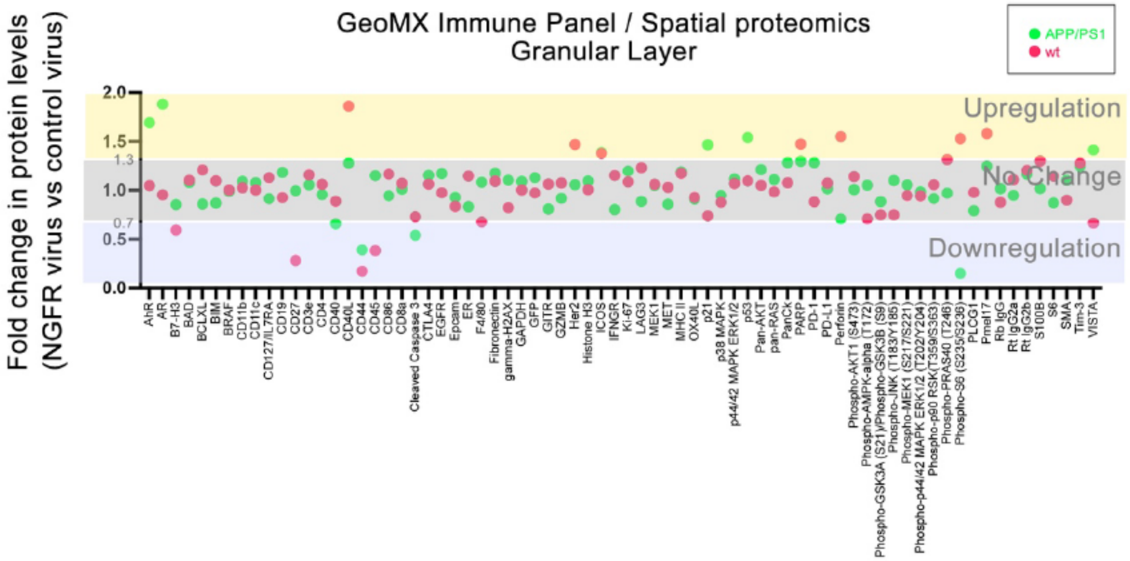

Supplementary Fig. 8. GeoMX neuro and immune panel spatial proteomics plot showing fold changes of protein targets in Ngfr-transduced brains versus control virus-transduced brains. Plots indicate targets and their fold changes in wild type animals (red dots) and APP/PS1dE9 mouse model of Alzheimer's disease (green dots). 1.3 and 0.7 are taken as upper and lower boundaries for the no-change criterion. **a.** Neuro panel. **b.** Immune panel.

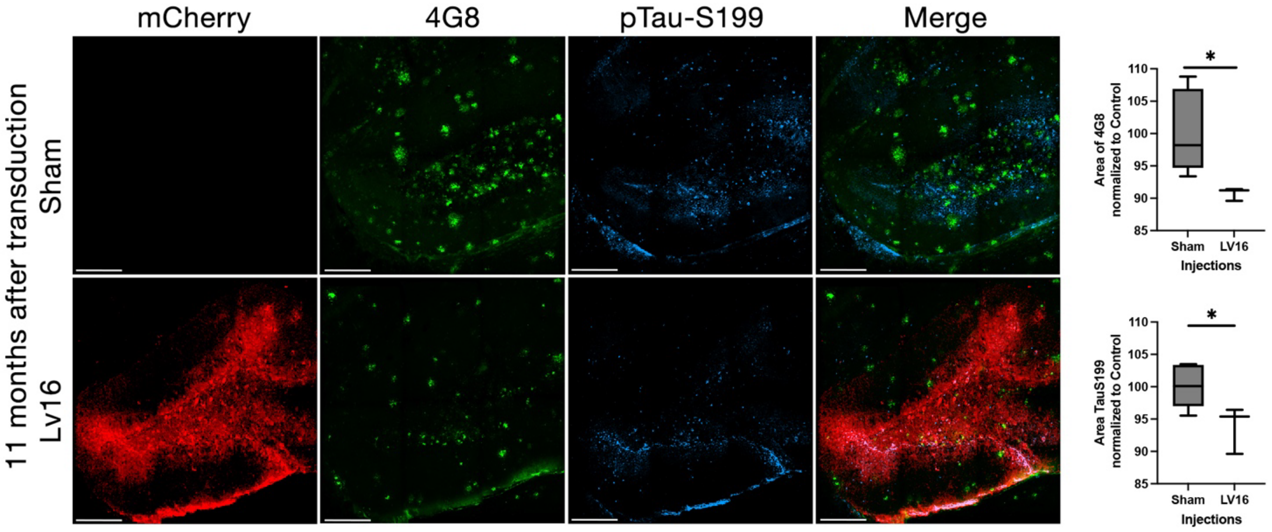

Supplementary Fig. 9. 11 months after transduction. APP/PS1dE9 mouse brain section immunostained for 4G8, mCherry and pTau-S199 with DAPI counterstain at 11 months after transduction with Lv16. Quantification graphs for normalized area of 4G8 and pTau-S199. Error bars represent standard error of the means.

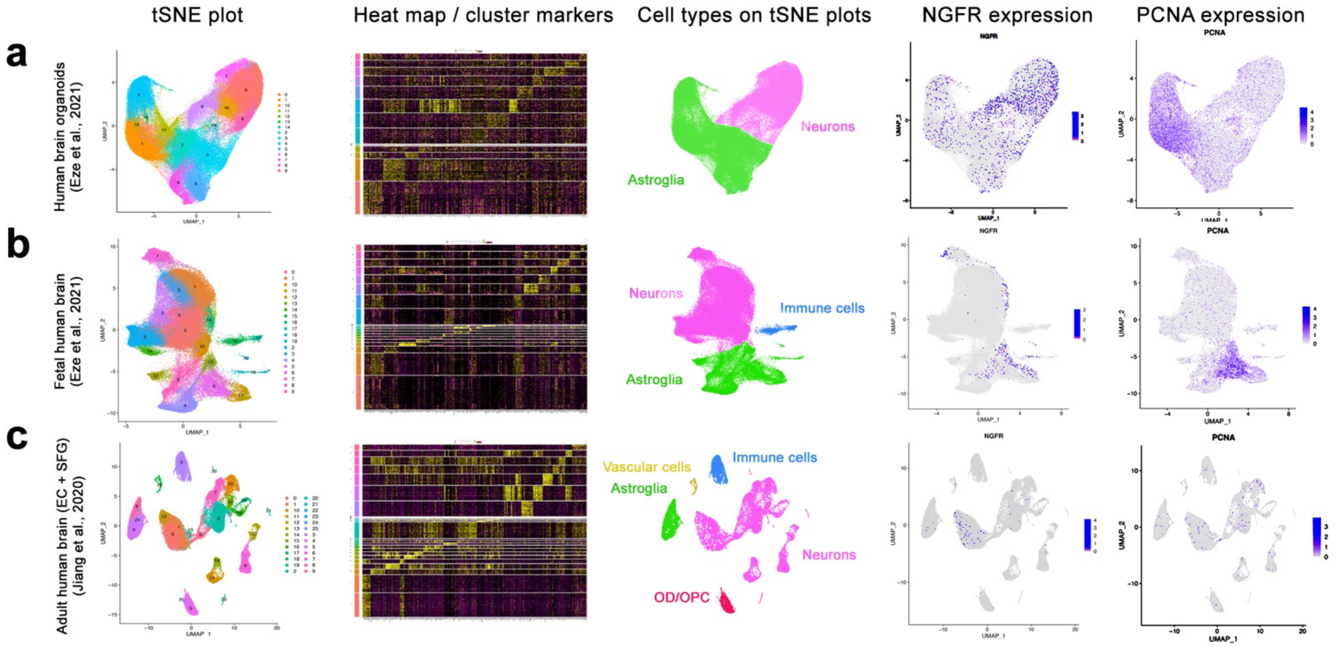

Supplementary Fig. 10. Single cell sequencing analyses of human brain organoids, fetal and adult human brains. tSNE plots for cell clustering, heat maps of top marker genes, identified cell types on colored tSNE plots, tSNE plot for *NGFR* and *PCNA* are shown for human datasets of **a.** human brain organoids, **b.** fetal human brain, and **(c)** adult human brain entorhinal cortex (EC) and superior frontal gyrus (SFG).

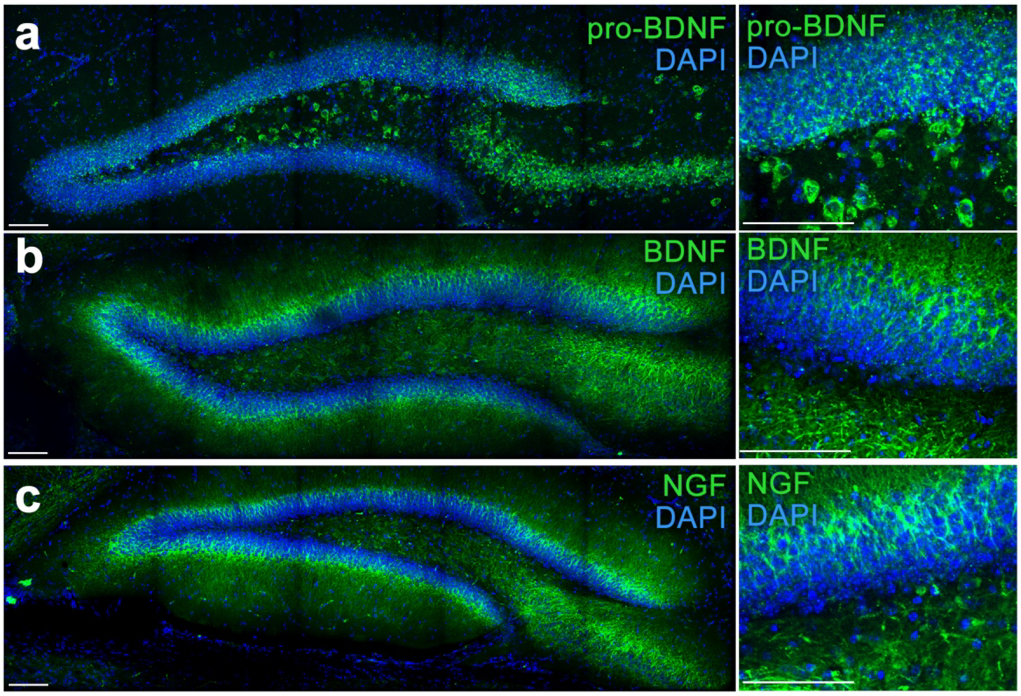

Supplementary Fig. 11. Expression of Ngfr ligands in mouse hippocampus. a-c. Expression of pro-BDNF, BDNF and NGF in mouse dentate gyrus. Panels on the right are high magnification from left panels. **a.** pro-BDNF, **b.** BDNF, **c.** NGF. BDNF and NGF are expressed in outer layers of DG, while pro-BDNF is expressed in the subgranular zone and hilus. Scale bars equal 25 μm.

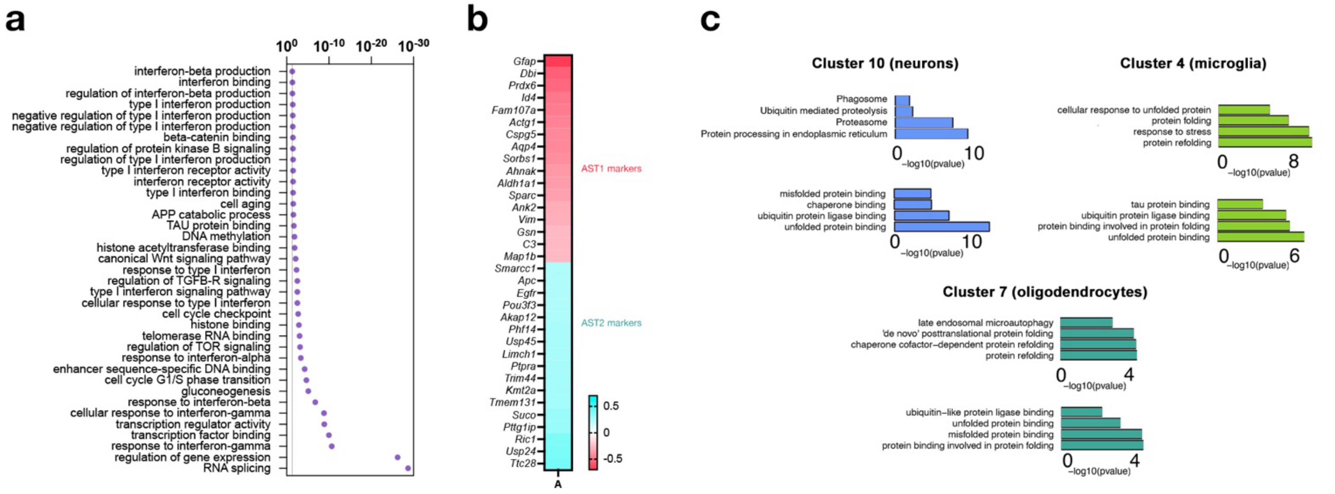

Supplementary Fig. 12. Potential mechanism of action downstream to Ngfr signalling. a. Enriched GO-terms after Ngfr signalling in astroglia in mouse DG. Interferon signalling-related terms are enriched. **b.** Heatmap of changes of genes associated with human reactive astroglia (AST1) and astroglia with neural progenitor activity (AST2). Ngfr reduces reactive astrocyte markers while increasing the progenitor cell markers. **c.** Differentially expressed genes after *Ngfr* transduction enrich the pathways related to enhanced protein quality control, toxic protein removal, misfolded protein response in non-neuronal cells. Selected GO-terms and KEGG pathways are depicted.

- **Supplementary Tables**

Supplementary Table 1. All materials and reagents used in this study. Provided online as .xlsx file.

Supplementary Table 2. Demographics of human brain samples used in this study. Provided online as .xlsx file.

- **Supplementary Data**

Supplementary Data 1. Differentially expressed genes in cell clusters of mouse brain after transduction of *Ngfr*. Provided online as .xlsx file.

Supplementary Data 2. KEGG pathway and GO term analyses on the genes that are downregulated in Cluster 6 (astroglia) after Lv16 transduction compared to control. Provided online as .xlsx file.

Supplementary Data 3. KEGG pathway and GO term analyses on the genes that are upregulated in Cluster 6 (astroglia) after Lv16 transduction compared to control. Provided online as .xlsx file.

Supplementary Data 4. All raw data that is used for statistical calculations. Tabs indicate figure panels. Provided online as .xlsx file.

Supplementary Data 5. Differential gene expression in astrocytes that are transduced with Ngfr virus versus non-transduced. Provided online as .xlsx file.

Supplementary Data 6. Differential gene expression in astrocytes that are transduced with Ngfr virus versus transduced with control virus. Provided online as .xlsx file.

Supplementary Data 7. Nanostring GeoMX spatial proteomics neuro panel raw data. Provided online as .xlsx file.

Supplementary Data 8. Nanostring GeoMX spatial proteomics immune panel raw data Provided online as .xlsx file.

Supplementary Data 9. Comparison of Data S6 to human ROSMAP AD cohort and zebrafish. Provided online as .xlsx file.

Supplementary Data. 10. Significant differential gene expression (DGE) (FDR < 0.05) in the AMP-AD datasets; CellCODE, PSEA, WLC analyses for cell intrinsic DGE from 3 different datasets (Mayo, MSSM, ROSMAP); WGCNA networks constructed from Mayo CER and TCX dataset. Provided online as .xlsx file.
