# Supplementary material for: Nerve growth factor receptor (Ngfr) induces neurogenic plasticity by suppressing reactive astroglial Lcn2/Slc22a17 signaling in Alzheimer’s disease
Source: NPJ Regen Med. 2023 Jul 10;8:33. doi: 10.1038/s41536-023-00311-5 (PMC10333226; doi:10.1038/s41536-023-00311-5)
Supplement: Supplementary file 4 — Reporting Summary [file 41536_2023_311_MOESM4_ESM.pdf]

nature portfolio

Corresponding author(s): Caghan Kizil

Last updated by author(s): May 13, 2023

Reporting Summary

Nature Portfolio wishes to improve the reproducibility of the work that we publish. This form provides structure for consistency and transparency in reporting. For further information on Nature Portfolio policies, see our Editorial Policies and the Editorial Policy Checklist.

Statistics

For all statistical analyses, confirm that the following items are present in the figure legend, table legend, main text, or Methods section.

n/a	Confirmed	
☐	☒	The exact sample size (*n*) for each experimental group/condition, given as a discrete number and unit of measurement
☐	☒	A statement on whether measurements were taken from distinct samples or whether the same sample was measured repeatedly
☐	☒	The statistical test(s) used AND whether they are one- or two-sided<br>*Only common tests should be described solely by name; describe more complex techniques in the Methods section.*
☐	☒	A description of all covariates tested
☐	☒	A description of any assumptions or corrections, such as tests of normality and adjustment for multiple comparisons
☐	☒	A full description of the statistical parameters including central tendency (e.g. means) or other basic estimates (e.g. regression coefficient) AND variation (e.g. standard deviation) or associated estimates of uncertainty (e.g. confidence intervals)
☐	☒	For null hypothesis testing, the test statistic (e.g. *F*, *t*, *r*) with confidence intervals, effect sizes, degrees of freedom and *P* value noted<br>*Give P values as exact values whenever suitable.*
☒	☐	For Bayesian analysis, information on the choice of priors and Markov chain Monte Carlo settings
☒	☐	For hierarchical and complex designs, identification of the appropriate level for tests and full reporting of outcomes
☒	☐	Estimates of effect sizes (e.g. Cohen's *d*, Pearson's *r*), indicating how they were calculated

Our web collection on statistics for biologists contains articles on many of the points above.

Software and code

Policy information about availability of computer code

Data collection	Spinning Disc Zeiss Axio Observer.Z1 microscope (Oberkochen, Germany) equipped with ZEN software (version blue edition, v3.2, company, Carl Zeiss, Jena, Germany), Operetta CLS microscope, at 20x magnification, GeoMx Digital Spatial Profiler (NanoString Technologies, Inc, Seattle, USA).
Data analysis	Fiji/ImageJ ver. 1.53, Zen Blue, Arivis (Vision 4D) were used to analyze the images as described in the methods section and Supplementary Table 1. Prism ver9.5 was used to analyze data and generate plots. R and Seurat were used to analyze the single-cell sequencing data and generating plots.

For manuscripts utilizing custom algorithms or software that are central to the research but not yet described in published literature, software must be made available to editors and reviewers. We strongly encourage code deposition in a community repository (e.g. GitHub). See the Nature Portfolio guidelines for submitting code & software for further information.

Data

Policy information about availability of data

All manuscripts must include a data availability statement. This statement should provide the following information, where applicable:

- Accession codes, unique identifiers, or web links for publicly available datasets
- A description of any restrictions on data availability
- For clinical datasets or third party data, please ensure that the statement adheres to our policy

All the datasets that support the findings of this study are available from the corresponding author on reasonable request.

Research involving human participants, their data, or biological material

Policy information about studies with human participants or human data. See also policy information about sex, gender (identity/presentation), and sexual orientation and race, ethnicity and racism.

Reporting on sex and gender	Amp-AD data can be accessed via the AD Knowledge Portal. For the AMP-AD accession numbers, please use the following IDs. Bulk DEGs: ROSMAP: syn3219045 (https://www.synapse.org/ #!Synapse:syn3219045); Mayo: syn5550404 (https://www.synapse.org/#!Synapse:syn5550404); MSBB: syn3159438 (https://www.synapse.org/#!Synapse:syn3159438). CI-DEG: syn22228843 (https://www.synapse.org/#!Synapse:syn22228843); WGCNA: syn5550404 (https://www. synapse.org/#!Synapse:syn5550404). The AD Knowledge Portal is a platform for accessing data, analyses and tools generated by the Accelerating Medicines Partnership (AMP-AD) Target Discovery Program and other National Institute on Aging (NIA)-supported programs to enable open- science practices and accelerate translational learning. The data, analyses and tools are shared early in the research cycle without a publication embargo on secondary use. Data is available for general research use according to the following requirements for data access and data attribution (https://adknowledgeportal.synapse.org/DataAccess/Instructions).
Reporting on race, ethnicity, or other socially relevant groupings	*Please specify the socially constructed or socially relevant categorization variable(s) used in your manuscript and explain why they were used. Please note that such variables should not be used as proxies for other socially constructed/relevant variables (for example, race or ethnicity should not be used as a proxy for socioeconomic status).*<br>*Provide clear definitions of the relevant terms used, how they were provided (by the participants/respondents, the researchers, or third parties), and the method(s) used to classify people into the different categories (e.g. self-report, census or administrative data, social media data, etc.)*<br>*Please provide details about how you controlled for confounding variables in your analyses.*
Population characteristics	The results published here are in whole or in part based on data obtained from the AMP-AD Knowledge Portal (doi:10.7303/syn2580853). Mayo Clinic: The Mayo RNAseq study data was led by Dr. Nilüfer Ertekin-Taner, Mayo Clinic, Jacksonville, FL as part of the multi-PI U01 AG046139 (MPIs Golde, Ertekin-Taner, Younkin, Price). Samples were provided from the following sources: The Mayo Clinic Brain Bank and Banner Sun Health Research Institute. Data collection was supported through funding by NIA grants P50 AG016574, R01 AG032990, U01 AG046139, R01 AG018023, U01 AG006576, U01 AG006786, R01 AG025711, R01 AG017216, R01 AG003949, NINDS grant R01 NS080820, CurePSP Foundation, and support from Mayo Foundation. Study data includes samples collected through the Sun Health Research Institute Brain and Body Donation Program of Sun City, Arizona. The Brain and Body Donation Program is supported by the National Institute of Neurological Disorders and Stroke (U24 NS072026 National Brain and Tissue Resource for Parkinson's Disease and Related Disorders), the National Institute on Aging (P30 AG19610 Arizona Alzheimer's Disease Core Center), the Arizona Department of Health Services (contract 211002, Arizona Alzheimer's Research Center), the Arizona Biomedical Research Commission (contracts 4001, 0011, 05-901 and 1001 to the Arizona Parkinson's Disease Consortium) and the Michael J. Fox Foundation for Parkinson's Research. MSBB: These data were generated from postmortem brain tissue collected through the Mount Sinai VA Medical Center Brain Bank and were provided by Dr. Eric Schadt from Mount Sinai School of Medicine. ROSMAP: Study data were provided by the Rush Alzheimer's Disease Center, Rush University Medical Center, Chicago. Data collection was supported through funding by NIA grants P30AG10161 (ROS), R01AG15819 (ROSMAP; genomics and RNAseq), R01AG17917 (MAP), R01AG30146, R01AG36042 (5hC methylation, ATACseq), RC2AG036547 (H3K9Ac), R01AG36836 (RNAseq), R01AG48015 (monocyte RNAseq) RF1AG57473 (single nucleus RNAseq), U01AG32984 (genomic and whole exome sequencing), U01AG46152 (ROSMAP AMP-AD, targeted proteomics), U01AG46161(TMT proteomics), U01AG61356 (whole genome sequencing, targeted proteomics, ROSMAP AMP-AD), the Illinois Department of Public Health (ROSMAP), and the Translational Genomics Research Institute (genomic). Additional phenotypic data can be requested at www.radc.rush.edu.
Recruitment	Not applicable for this study.
Ethics oversight	https://www.nia.nih.gov/research/amp-ad. The analyses conducted at Mayo Clinic were approved by the appropriate Mayo Clinic Institutional Review Board. Additionally, Human brain samples were obtained from New York Brain Bank within institutional regulations of Columbia University.

Note that full information on the approval of the study protocol must also be provided in the manuscript.

Field-specific reporting

Please select the one below that is the best fit for your research. If you are not sure, read the appropriate sections before making your selection.

☒ Life sciences ☐ Behavioural & social sciences ☐ Ecological, evolutionary & environmental sciences

Life sciences study design

All studies must disclose on these points even when the disclosure is negative.

Sample size	The planned sample size was motivated by a power analysis conducted with G*power.
Data exclusions	No data points were excluded from analysis unless indicated.
Replication	All experiments included a sufficient sample size, taking into account the expected variability for in vivo and in vitro conditions. Representative data were confirmed at least once with an independent experiment.
Randomization	Animals were assigned randomly to experimental and control groups.
Blinding	Blinding was used during analysis where manual/semi-automated/automated methods were used to quantify different cell types/ fluorescence intensties.

Reporting for specific materials, systems and methods

We require information from authors about some types of materials, experimental systems and methods used in many studies. Here, indicate whether each material, system or method listed is relevant to your study. If you are not sure if a list item applies to your research, read the appropriate section before selecting a response.

Materials & experimental systems

n/a	Involved in the study	
☐	☒	Antibodies
☐	☒	Eukaryotic cell lines
☒	☐	Palaeontology and archaeology
☐	☒	Animals and other organisms
☐	☒	Clinical data
☒	☐	Dual use research of concern
☒	☐	Plants

Methods

n/a	Involved in the study	
☒	☐	ChIP-seq
☒	☐	Flow cytometry
☒	☐	MRI-based neuroimaging

Antibodies

Antibodies used

Primary Antibodies
4G8 (anti-β-amyloid) Biolegend 80070
Aldoc Abcam ab87122
AT8 ThermoFisher MN1020
BDNF Alomone labs ANT-010
Beta-III-tubulin R&D MAB1195
BrdU BIO-RAD MCA2060
Dcx Abcam ab207175
Gfap Invitrogen A-21282
Gfap Abcam ab7260
Gfap ThermoFisher OPA1-06100
Hopx Santa Cruz sc-398703
Iba1 Wako 019-19741
Lcn2 R&D system AF1857
Lcn2 ThermoFisher MA5-41651
Map2 Sigma M4403-.2ML
mCherry Abcam ab205402
Mcm7 Santa Cruz sc-9966
NeuN Abcam ab177487
Ngf Sigma-Aldrich AB1526
Ngfr Origene TA328682
Olig2 Mybiosource MBS502172
proBdnf Alomone labs ANT-006
pTau-S199 Invitrogen 44-734G
S100β Dako z0311
Slc22A17 Sigma-Aldrich SAB3500306
Secondary Antibodies
Goat anti-chicken IgY,Alexa 488 Thermo Fischer Scientific A11039
Goat anti-chicken IgY, Alexa 555 Thermo Fischer Scientific A21437
Goat anti-chicken IgY, Alexa 647 Thermo Fischer Scientific A21449
Goat anti-mouse IgG1, Alexa 488 Thermo Fischer Scientific A21121

	Goat anti-mouse IgG1, Alexa 555 Thermo Fischer Scientific A21127<br>Goat anti-mouse IgG1, Alexa 647 Thermo Fischer Scientific A21240<br>Goat anti-mouse IgG2b, Alexa 488 Thermo Fischer Scientific A21141<br>Goat anti-mouse IgG2b, Alexa 555 Thermo Fischer Scientific A21147<br>Goat anti-mouse IgG2b, Alexa 647 Thermo Fischer Scientific A21242<br>Goat anti-rabbit IgG, Alexa 488 Thermo Fischer Scientific A21428<br>Goat anti-rabbit IgG, Alexa 555 Thermo Fischer Scientific A21428<br>Goat anti-rabbit IgG, Alexa 647 Thermo Fischer Scientific A21245<br>Goat anti-rat IgG, Alexa 488 Thermo Fischer Scientific A11006<br>Goat anti-rat IgG, Alexa 555 Thermo Fischer Scientific A21434<br>Goat anti-rat IgG, Alexa 647 Thermo Fischer Scientific A21247<br>Donkey anti-goat, Alexa 488 Thermo Fischer Scientific A11055<br>Donkey anti-goat, Alexa 555 Thermo Fischer Scientific A21432
Validation	Antibody registry ID of each antibody is as follows:<br>4G8 (anti-β-amyloid) -AB_2564633<br>Aldoc -AB_10673854<br>AT8 -AB_223647<br>BDNF -AB_2039756<br>Beta-III-tubulin -AB_357520<br>BrdU -AB_323427<br>DCX -AB_2894710<br>GFAP -AB_2535827<br>GFAP -AB_305808<br>GFAP -AB_325657<br>Hopx -AB_2687966<br>Iba1 -AB_839504<br>LCN2 -AB_355022<br>LCN2 -AB_2899133<br>mCherry -AB_2722769<br>Map2 -AB_477193<br>Mcm7 -AB_627235<br>NeuN -AB_2532109<br>NGF -AB_90733<br>NGFR -validated by Origene<br>Olig2 -AB_2157543<br>proBDNF -AB_2039758<br>pTau-S199 -AB_2533737<br>S100β -AB_10013383<br>Slc22A17 -AB_10639344<br>All secondary antibodies were validated by ThermoFischer

Eukaryotic cell lines

Policy information about cell lines and Sex and Gender in Research

Cell line source(s)	Primary human astrocytes (ScienCell Research Laboratory -1800)<br>HEK293T cells (ATCC CRL-3216)<br>Mouse neural stem/progenitor cells (mNSPCs) (Mashkaryan, et. al. 2020)<br>Lv16-p6NST90-hUb-mNGFR-T2A-mCherry human astrocytes> This paper<br>Lv13-p6NST90-hUb-mCherry human astrocytes >This paper<br>Lv16-p6NST90-hUb-mNGFR-T2A-mCherry mouse NSPCs >This paper<br>Lv13-p6NST90-hUb-mCherry mouse NSPCs >This paper
Authentication	Primary human astrocytes validated by ScienCell Research Laboratory<br>HEK293T cells validated by ATCC CRL<br>Mouse neural stem/progenitor cells (mNSPCs) previously reported by Mashkaryan, et. al. 2020<br>All cell lines generated during this study were validated using different antibody staning and functional assays.
Mycoplasma contamination	No indication of contamination was observed.
Commonly misidentified lines<br>(See ICLAC register)	No commonly misidentified cell lines were used.

Animals and other research organisms

Policy information about studies involving animals; ARRIVE guidelines recommended for reporting animal research, and Sex and Gender in Research

Laboratory animals	Wildtype and B6.Cg-Tg(APP695)3DboTG(PSEN1dE)mice (both male and female) age 52-56 weeks (age exceptions are mentioned for each individual case) were used during this study.

Wild animals	No wild animals were used in this study.
Reporting on sex	Equal number of sexes were used. Findings are not associated to sex.
Field-collected samples	No field-collected samples were used in this study.
Ethics oversight	All animal experiments were performed in accordance with the applicable European regulations and approved by the responsible authority (Landesdirektion Sachsen Germany and TU Dresden-Kommission für Tierversuche) under license number TVV 87/2016. Animals were handled with extreme precaution to reduce suffering and overall animal numbers. Human brain samples were obtained from New York Brain Bank within institutional regulations of Columbia University. The analyses conducted at Mayo Clinic were approved by the appropriate Mayo Clinic Institutional Review Board.

Note that full information on the approval of the study protocol must also be provided in the manuscript.

Clinical data

Policy information about clinical studies

All manuscripts should comply with the ICMJE guidelines for publication of clinical research and a completed CONSORT checklist must be included with all submissions.

Clinical trial registration	*Provide the trial registration number from ClinicalTrials.gov or an equivalent agency.*
Study protocol	*Note where the full trial protocol can be accessed OR if not available, explain why.*
Data collection	*Describe the settings and locales of data collection, noting the time periods of recruitment and data collection.*
Outcomes	*Describe how you pre-defined primary and secondary outcome measures and how you assessed these measures.*
